# A Review on the Partial and Complete Dissolution and Fractionation of Wood and Lignocelluloses Using Imidazolium Ionic Liquids

**DOI:** 10.3390/polym12010195

**Published:** 2020-01-11

**Authors:** Hatem Abushammala, Jia Mao

**Affiliations:** 1 Fraunhofer Institute for Wood Research (WKI), Bienroder Weg 54E, 38108 Braunschweig, Germany; 2 Department of Mechanical Engineering, Al-Ghurair University, Dubai International Academic City, Dubai P.O. Box 37374, UAE; jia.mao@agu.ac.ae

**Keywords:** cellulose, wood, lignocellulose, ionic liquid, imidazolium, fractionation, dissolution

## Abstract

Ionic liquids have shown great potential in the last two decades as solvents, catalysts, reaction media, additives, lubricants, and in many applications such as electrochemical systems, hydrometallurgy, chromatography, CO_2_ capture, etc. As solvents, the unlimited combinations of cations and anions have given ionic liquids a remarkably wide range of solvation power covering a variety of organic and inorganic materials. Ionic liquids are also considered “green” solvents due to their negligible vapor pressure, which means no emission of volatile organic compounds. Due to these interesting properties, ionic liquids have been explored as promising solvents for the dissolution and fractionation of wood and cellulose for biofuel production, pulping, extraction of nanocellulose, and for processing all-wood and all-cellulose composites. This review describes, at first, the potential of ionic liquids and the impact of the cation/anion combination on their physiochemical properties and on their solvation power and selectivity to wood polymers. It also elaborates on how the dissolution conditions influence these parameters. It then discusses the different approaches, which are followed for the homogeneous and heterogeneous dissolution and fractionation of wood and cellulose using ionic liquids and categorize them based on the target application. It finally highlights the challenges of using ionic liquids for wood and cellulose dissolution and processing, including side reactions, viscosity, recyclability, and price.

## 1. Introduction

The dependency on fossil feedstock and the current consumption style are global challenges that triggered the search for sustainable processes for the efficient utilization of renewable resources such as biomass [1,2]. Lignocellulosic biomass is the Earth’s most abundant renewable resource, which includes forest and food crops, their harvesting residues, and industrial and municipal lignocellulosic waste [3,4]. In Europe, hundreds of millions of tons of lignocellulosic residues are generated every year, most of which are underutilized, burnt, or end up in dumping sites releasing greenhouse gases into the atmosphere [5]. In more detail, around 80 million tons of forest residues, 350 million tons of crop residues, 90 million tons of industrial lignocellulosic residues, and 200 million tons of municipal lignocellulosic waste are generated every year in Europe, one-third of which is available for utilization [6]. In Germany, for instance, a fraction (ca. 2–3 million tons/year) of saw-rest wood as a residue is processed into pellets, which are burnt as a source of bioenergy [7].

Other than the urgent need for utilizing biomass and bio-residues, there is also the need to develop green technologies for processing these massive amounts to bio-based materials and products. Wood and its polymers are insoluble in most of the common industrial solvents due to the strong intermolecular and intramolecular networks of hydrogen bonds and complex microstructure [8,9]. For cellulose processing, carbon disulfide (toxic), N-methylmorpholine-N-oxide (more environmentally acceptable), and dimethylsulfoxide (DMSO), dimethylformamide (DMF), and dimethylacetamide (DMAc) in combination with lithium chloride are used [10,11]. However, there is still a need to develop more environmentally friendly solvents for cellulose and wood processing.

In the 1990s, ionic liquids were proposed as “green solvents” for wood and cellulose dissolution. They are salts that are liquids at temperatures below 100 °C. They have unique properties such as low vapor pressure, high thermal and chemical stability, non-flammability, chemical tunability, and a broad electrochemical window [12,13,14]. The low vapor pressure of ionic liquids, which is a result of the strong interaction forces between their constituting ions, made them considered green solvents in general as they do not emit potentially hazardous organic compounds during use, handling, and transportation [15]. They also have a wide range of solvation power and selectivity by simply tuning the chemistry of the ions they are made of. They are, therefore, excellent substitutes for volatile organic solvents in chemical processes. Other than their potential as solvents, they have also shown great potential in a wide range of applications, including electrochemical systems, energy-harvesting devices, chromatography, lubrication, chemical catalysis, CO_2_ capture, and hydrometallurgy [16,17,18,19,20].

**Figure 1:**
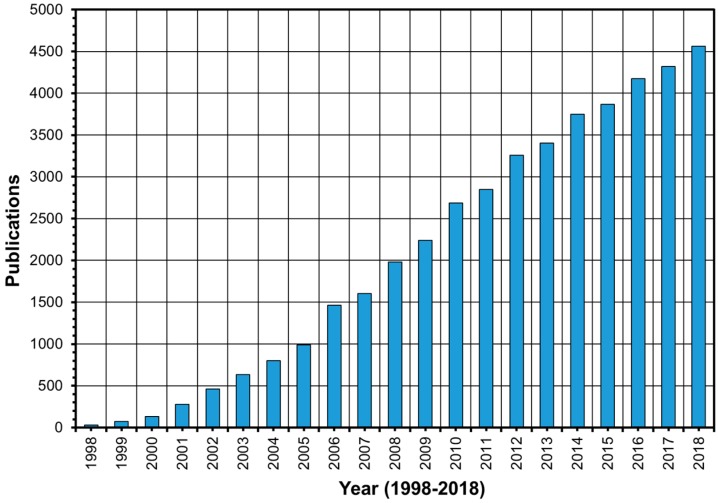
The number of publications in the last two decades showing the increasing interest in ionic liquids (Web of Science, November 2019, ionic liquids).

The first report on ionic liquids goes back to 1914 by Paul Walden, who prepared the ionic liquid ethylammonium nitrate [21]. They did not, however, receive much attention until 1999, before which there was less than 35 publications on ionic liquids per year compared to more than 4500 in 2018 (Figure 1). Ionic liquids production has also been industrialized by many companies such as BASF (Germany), Acros Organics (Belgium), EMD (USA), Iolitec (Germany), Sigma-Aldrich (Germany), and TCI Chemicals (Japan). The market of ionic liquids is estimated to be 2.2 billion US dollars in 2022 and to continue to grow [22]. The first report on the use of ionic liquid for cellulose processing is thought to be in 2002 by Swatloski et al., who explored the solubility of pulp cellulose in different ionic liquids [23]. However, two patents in 1933 and 1934 by Graenacher claim the use of molten pyridinium and ammonium salts to dissolve cellulose [24,25]. On the other hand, the first report on wood dissolution using ionic liquids seems to go back to 2006 by Honglu and Tiejun, who dissolved the dawn redwood (softwood) using two imidazole-based ionic liquids and showed that ionic liquids could be better liquefaction agents than phenol/H_2_SO_4_, a common liquefaction agent at the time [26]. Few reports on wood and ionic liquids were published earlier to Honglu’s, which, however, investigated the potential of ionic liquids for wood preservation and electrostatic control [27,28,29]. More reports followed Honglu’s by Kilpelainen et al. and Fort et al. [30,31]. In the last five years (2014–2018), the literature was enriched by around 2000 publications on the use of ionic liquids for wood and cellulose dissolution and processing.

**Figure 2:**
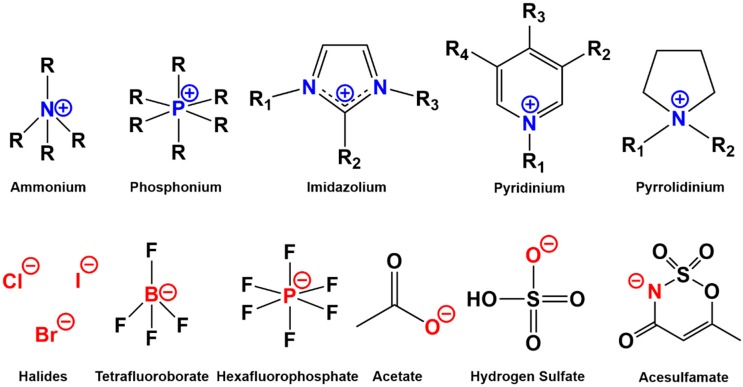
Some of the cations and anions that constitute most of the commonly used ionic liquids for wood and cellulose dissolution and fractionation.

Ionic liquids can almost be made of unlimited combinations of cations and anions, which are relatively less regular in size than those constituting simple salts such as sodium chloride (Figure 2) [32]. This results in crystalline structures with significantly lower lattice energies and melting points. The cations of most common ionic liquids varied from tetra-alkyl ammonium and tetra-alkyl phosphonium to imidazolium, pyridinium, and pyrrolidinium. Cations that are even more complex have been reported in the literature [33]. The anions also varied from simple halides, acetate, nitrate, hydrogen sulfate to more complicated anions such as acesulfamate [34]. As a result, it is possible to synthesize ionic liquids with a specific melting point, density, viscosity, hydrophilicity, and electrical conductivity to be used for a certain application or chemical process [35,36]. For the processing of wood, lignocellulose, and cellulose, imidazolium ionic liquids have been the most commonly used, which are all based on 1-alkyl-3-alkylimidazolium as a cation (Table 1). 

The impact of the constituting ions on the physicochemical properties of ionic liquids is significant. For instance, [BMIM][Cl] and [EMIM][Cl] are solid at room temperature while [BMIM][OAc] and [EMIM][OAc] are liquid. [BMIM][BF_4_] and [EMIM][BF_4_] are liquid at room temperature while [BnMIM][BF_4_] is solid. In terms of viscosity, [BMIM][HSO_4_] has a viscosity of ca. 3100 cP compared to ca. 1500 cP for [EMIM][HSO_4_] due to the higher van der Waal forces for longer alkyl chains [37,38]. Both are significantly more viscous than [BMIM][PF_6_] and [EMIM][PF_6_], [BMIM][BF_4_], and [EMIM][BF_4_], respectively. A study on [BMIM] ionic liquids with carboxylates of different chain lengths (formate, acetate, propionate, and butyrate) showed that increasing the chain length significantly increases the viscosity of the ionic liquid [39]. It is important to mention here that the values of melting temperature and viscosity are strongly affected by the presence of water and impurities. This is the reason behind the variation in the melting point and viscosity values in the literature. The density of imidazolium ionic liquids ranged between 1.0 to 1.4 g/cm^3^, which is slightly higher than that for common organic solvents (0.7–1.0 g/cm^3^) [40]. It is strongly dependent on the molecular mass of the cation and anion. For instance, the density of [BMIM][PF_6_] is higher than [BMIM][BF_4_] and they are both higher than [BMIM][Cl], [BMIM][Br], and [BMIM][OAc] [41]. The thermal stability of imidazolium ionic liquids is strongly dependent on the anion. The degradation temperature varies between 200 to 500 ºC and increases in the following order [PF_6_] > [BF_4_] > [Cl] = [Br] = [I] [42]. Minor changes in the degradation temperature were reported between [EMIM] and [BMIM] [43].

Imidazolium ionic liquids may exhibit acid/base properties, which play an important role in their performance. [BMIM][HSO_4_] is an acidic ionic liquid while [BMIM][OAc] is basic due to the acidity of hydrogen sulfate and the basicity of acetate, respectively [44,45]. The acidity/basicity of imidazolium ionic liquids strongly depends on the acidity/basicity of the anion. For instance, [BMIM][OAc] is significantly more basic than [BMIM][Cl] because the acetate anion is more basic than chloride while [EMIM][OAc] showed slightly higher basicity than [BMIM][OAc] [46].

**Table 1:** Summary of the most commonly used imidazolium ionic liquids for wood and cellulose dissolution and fractionation and their physicochemical properties.

Ionic Liquid	Properties				Ref.
	Melting Temp. (°C)	Density (g/cm^3^) at 25 °C	Viscosity (cP) at 20–30 °C	Electrical Conduct. (mS/cm) at 25 °C	
[BMIM][OAc]	−20	1.1	208	1.4	[47,48]
[BMIM][Cl]	41–70	1.1	Solid	-	[47,48]
[BMIM][Br]	60–81	1.1	Solid	-	[47,48,49]
[BMIM][I]	−72	1.4–1.5	1110–1183	0.5	[47,48]
[BMIM][HSO_4_]	-	1.3	3088	-	[47,48]
[BMIM][BF_4_]	−83–−74	1.1–1.3	72−233	3.2	[47,48]
[BMIM][PF_6_]	11	1.3–1.4	207–450	1.5–4.8	[47,48]
[BMIM][Ace]	30	1.2	800	0.5	[50]
[EMIM][OAc]	−45–−14	1.0–1.1	91–162	2.5–2.8	[47,48]
[EMIM][Cl]	80–89	1.1–1.2	Solid	-	[47,48]
[EMIM][Br]	65–91	-	Solid	-	[47,48]
[EMIM][I]	79–85	-	Solid	-	[47,48]
[EMIM][HSO_4_]	-	1.4	1510	0.5	[48]
[EMIM][BF_4_]	6–15	1.2–1.4	34–66	13.0–14.1	[47,48]
[EMIM][PF_6_]	58–64	1.4	450	5.2	[47,48,49]
[EMIM][Ace]	34	1.3	556	0.6	[50]
[AMIM][Cl]	47	-	-	-	[48]
[AMIM][I]	57	-	-	-	[48]
[BnMIM][Cl]	75	-	Solid	-	[48]
[BnMIM][BF_4_]	78	-	Solid	-	[48]
[BnMIM][PF_6_]	130–135	-	Solid	-	[47,48]

[BMIM]: 1-butyl-3-methylimidazolium, [EMIM]: 1-ethyl-3-methylimdiazolium, [AMIM] 1-allyl-3-methylimidazolium, [BnMIM]: 1-benzyl-3-methylimidazolium, [OAc]: acetate, [Ace]: acesulfamate, [BF_4_]: tetrafluoroborate, [PF_6_]: hexafluorophospohate.

This review discusses the potential of ionic liquids with a focus on 1-alkyl-3-methylimidazium as cation for the dissolution and fractionation of wood and cellulose. The literature reports will be categorized based on the following target applications: (1) dissolution of wood and cellulose to overcome their recalcitrance to enzymatic hydrolysis for biofuel production, (2) fractionation of wood to its individual components before each being utilized in a certain application, (3) processing wood and cellulose to all-wood and all-cellulose composites, and (4) extraction of cellulose nanoparticles from wood and cellulose. This review will not cover the use of ionic liquids as solvents for the chemical modification of cellulose as it has been thoroughly reviewed [51,52].

## 2. Dissolution Capability and Selectivity of Imidazolium Ionic Liquids to Wood Polymers

The selection of a cation and anion combination does not only influence the physicochemical properties of ionic liquids but also their performance in certain applications and processes. With the focus on the dissolution and processing of wood and cellulose, it is of great importance to have an idea about the solvation power and selectivity of ionic liquids to wood polymers: cellulose, hemicelluloses, and lignin. Some studies relied on the solubility of the individual wood polymers in ionic liquids, while others compared the amount of each polymer that can be dissolved when the wood is treated with an ionic liquid [53]. Although it is believed that ionic liquids are able to dissolve wood and its polymers simply by disrupting the strong hydrogen bond networks between their molecular chains, the process is more complicated and may involve other processes such as hydrolysis and chemical derivatization. These side processes will be thoroughly discussed at a later stage in this review.

### 2.1. Impact of the Cation and Anion Combination

There are four imidazolium cations that are most commonly used for the dissolution of wood and cellulose: 1-ethyl-3-methylimidazolium ([EMIM]), 1-butyl-3-methylimidazolium ([BMIM]), 1-allyl-3-methylimidazolium ([AMIM]), and 1-benzyl-3-methylimidazolium ([BnMIM]). The main difference is the substituent at the N1 position at the imidazolium ring. Ionic liquids with no substituent at that position, i.e., [HMIM] where H stands for hydrogen, tend to be acidic [54]. Studies showed that [EMIM] and [BMIM] ionic liquids are better solvents for cellulose than [AMIM] and [BnMIM] ionic liquids due to the hydrophobicity introduced to the imidazolium ring by the allyl and the benzyl groups. On the other hand, [AMIM] and [BnMIM] are better lignin solvents due to the pi-pi interaction of the allyl group and benzyl group with the phenolic rings of lignin [55]. For the same reason, [BnMIM][Cl] can mainly dissolve lignin but not cellulose while [EMIM][Cl], [BMIM][Cl], and [AMIM][Cl] can dissolve all wood components [56]. [AMIM][Cl] is a better wood solvent than [EMIM][Cl] and [BMIM][Cl] because it better interacts with both lignin and cellulose, which supports it accessibility inside wood microstructure [30,57]. The same applies to [AMIM][OAc], [BMIM][OAc], and [EMIM][OAc] [58]. It has also been reported that [EMIM][OAc] can dissolve more cellulose but slightly less lignin than [BMIM][OAc] due to the higher hydrophobicity of butyl compared to ethyl, which is favored by lignin [59]. The same applies to [EMIM][Cl] and [BMIM][Cl] [60,61].

The chemistry of the anion has also a strong impact on the dissolution capability and selectivity of the ionic liquid [62]. Due to its high basicity, the acetate anion [OAc] can efficiently break hydrogen bonding and therefore allows better wood dissolution compared to chloride [Cl] [63]. It also showed a higher dissolution affinity to lignin than chloride [61,64]. The acetate ion is more capable of attacking the hydrogen of the β carbon of lignin inducing the cleaving of β-O-4 bonds in lignin [65]. The formate [OF] anion showed the same power of acetate to lignin dissolution but more power to the dissolution of hemicelluloses [58]. Due to its big size, the acesulfamate anion showed a complete selectivity to lignin dissolution because it hinders its diffusion into the crystalline regions of cellulose [50,66] while dicyanamide ionic liquids cannot dissolve wood or cellulose [62]. Unlike acetate and chloride ions, hydrogen sulfate is an acidic anion, which supports the hydrolysis of wood polysaccharides and lignin condensation in a behavior similar to sulfuric acid [44,67,68]. To better categorize the polarity and acidity/basicity of ionic liquids and foresee their interaction with wood polymers and other materials, their Kamlet–Taft parameters have been determined [69,70]. Finally, it is important to mention that part of the impact of the cation and anion on the interaction of ionic liquids with wood polymers is due to their influence on the viscosity of the ionic liquid. Ionic liquids with lower viscosities tend to have more solvation power to wood and cellulose.

### 2.2. Impact of the Addition of Water, Organic Solvents, and Salts

Ionic liquids are miscible with water and most organic solvents, and their mixtures have been explored for processing wood and cellulose. Some of these studied only aimed at reducing the viscosity of the ionic liquid while others investigated the impact of water or organic solvent addition on the properties of the ionic liquid and its dissolution power to wood polymers [71]. Fendt et al. have shown that the addition of 5% (w/w) of water or organic solvents (acetonitrile and ethylene glycol) to [EMIM][OAc] and [BMIM][OAc] decreased their viscosity by 50% [72]. Many reports showed that the presence of water decreased the solubility of cellulose in ionic liquids because the water supports the reformation of hydrogen bonding of cellulose and affects the cation/anion interactions [23,73,74,75]. Similar behavior was reported for some organic solvents [76]. On the other hand, water addition to ionic liquids facilitated lignin dissolution and depolymerization [77], hemicelluloses dissolution [78,79,80], and hydrolysis of the lignin-carbohydrate complex [81], which assisted the disintegration of wood. These observations, however, do not apply to DMSO, DMF, and DMAc, which reduced the viscosity of the ionic liquids and supported the dissolution of wood polymers, including cellulose [82,83,84,85]. They also supported the dissociation of the ionic liquid and increasing the concentration of the ions as a result [86]. Some studies tailored the dissolution power and selectivity to wood polymers by adjusting the pH of the ionic liquid [87,88] or by the addition of salts such as LiCl and Na_2_SiO_3_ [89,90,91].

### 2.3. Impact of Dissolution Conditions

It is not only the chemistry of the ionic liquid that determines the efficiency of wood dissolution. Process parameters, such as temperature and time have a significant influence [92]. Temperature does not only push the kinetics of the process forward but also reduces the viscosity of the ionic liquid, supporting further dissolution. Temperature also affects the selectivity of ionic liquids to wood polymers. For instance, many studies have reported the strong capability of [EMIM][OAc] to wood dissolution, most of which have used high dissolution temperatures and time. However, It has been shown that reducing the reaction severity allows the dissolution of the majority of lignin and hemicelluloses of wood with a minimum cellulose dissolution in a similar scenario to wood pulping [93]. On the other hand, [BMIM][Ace], which was proven not to be able to dissolve cellulose [50], could disintegrate cellulose at high dissolution temperatures (130 °C) [94].

Other process parameters such as stirring speed, solid/liquid ratio, wood and cellulose particle and molecular size [60,95,96], the use of microwave heating [97,98], and ultrasonication [99] are also important and have been thoroughly explored in the literature. It has also been observed that wood dissolution is affected by environmental conditions. For instance, humidity and oxygen supported the depolymerization of wood polymers in [EMIM][Cl] [100].

## 3. Fractionation and Regeneration of Lignocellulosic Polymers upon Dissolution in Ionic Liquids

**Figure 3:**
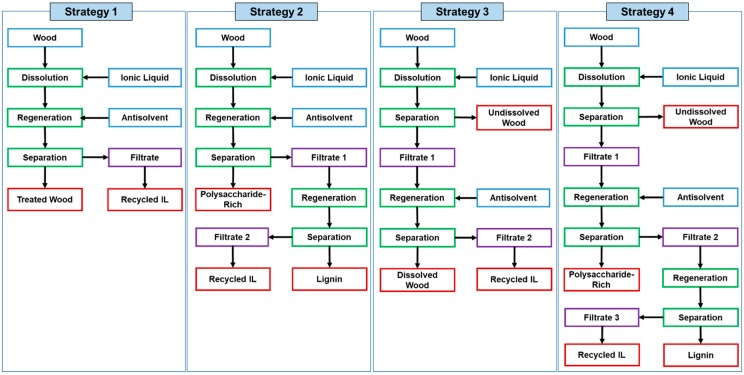
The different fractionation strategies followed in the literature upon partial or complete dissolution of wood or lignocelluloses.

Upon a complete (homogeneous) or partial (heterogonous) dissolution of wood, different strategies have been used to separate the dissolved and undissolved fractions, which can be summarized into four (Figure 3). In the first strategy, the wood is partially or completely dissolved, and the reaction mixture is then fully regenerated using an anti-solvent without any fractionation. The regenerated wood is collected by centrifugation or filtration, and the ionic liquid is then recycled. This strategy is mostly used for processing wood into composites, as there is no need for wood fractionation. It is also used when the main aim of dissolution is breaking the recalcitrant of wood for biofuel production [101]. In the second strategy, the wood is dissolved, mainly completely, and the dissolved wood is fractioned to polysaccharide-rich fraction and lignin fraction by selective regeneration. The polysaccharide-rich fraction is usually explored for biofuel production, as the removal of lignin facilitates the enzymatic hydrolysis of the polysaccharides to simple sugars. It can also be fractioned further to hemicelluloses and cellulose using alkaline hydrolysis. The third and fourth strategies are mostly used for a thorough fractionation of wood upon partial dissolution. The third strategy separates the dissolved wood from the undissolved using centrifugation or filtration, which is then regenerated completely with no fractionation resulting in two fractions: dissolved wood and undissolved. While the fourth strategy fractions the dissolved wood further to lignin and polysaccharide fractions resulting in three fractions: undissolved wood, polysaccharide-rich fraction, and lignin. The undissolved wood in both strategies could be processed further to pulp fibers or cellulose nanoparticles. The procedure to separate the dissolved wood from the undissolved depends on the viscosity of the reaction mixture. If the reaction is viscous as in the case of using ionic liquids with no dilution, centrifugation is usually used as filtration could be impossible to perform. Sometimes, a co-solvent such as DMSO, DMF, or DMAc is added to the reaction mixture upon dissolution to allow the separation of wood fractions by filtration [102,103].

The regeneration of the dissolved wood using antisolvents is a crucial process. The selection of anti-solvent affects the recovery of the dissolved wood and the efficiency of fractionation. When an anti-solvent is added to a reaction mixture, the ions of the ionic liquids are extracted into the liquid phase through hydrogen bonding and coulombic forces shielding them from direct interaction with wood polymers. This also disrupts the cation/anion solvation network triggering the hydrogen bonds to reform [104,105]. Water is the most used antisolvent for complete regeneration of wood with no fractionation. Methanol and ethanol have also been used [106,107]. To fraction the dissolved wood to a polysaccharide-rich fraction and lignin fraction, a variety of antisolvents has been used. Among these, acetone:water (Ac/W) mixtures are the most commonly used [108] due to the easiness of tailoring its polarity by controlling the Ac/W mixing ratio and by evaporating the acetone. When added to dissolved wood, the polysaccharides (with some lignin) are regenerated while keeping lignin soluble in the mixture. The acetone is then evaporated, increasing the polarity of the mixture and triggering lignin to precipitate, which can then be isolated. The efficiency of the fractioning process and the purity of the polysaccharide fraction are higher using high Ac/W ratios. Unfortunately, Ac/W mixtures with high Ac\W ratios (2:1 and more) could be immiscible with some ionic liquids such as [EMIM][OAc]. A ratio of 9:1 is recommended for [BMIM][Cl] and 1:1 for [EMIM][OAc] [108,109]. In some reports, Ac/W-based regeneration is differently performed. The acetone is added at first to precipitate the polysaccharides, then water is added to precipitate lignin [110]. The same approach was used using ethanol instead of acetone [96,111]. A solution of sodium hydroxide (usually 0.1M) was also used as an antisolvent for wood fractionation [112]. When added to a dissolved wood mixture, the polysaccharides (with some lignin) are regenerated and separated. The IL/antisolvent mixture is then acidified to a pH of 2 to regenerate lignin [97,113].

## 4. Applications of Ionic Liquids for Lignocellulose and Cellulose Dissolution and Processing

### 4.1. Wood and Cellulose Dissolution for Biofuel Production

Ionic liquids have received a significant attention because of their effectiveness in reducing the recalcitrance of biomass to enzymatic hydrolysis towards the production of bioethanol [114]. Ionic liquids also reduce the need for corrosive and toxic chemicals and the accompanying waste streams that are usually generated with alkaline and acidic reagents [115,116]. An ionic liquid-mediated pretreatment of wood aims at effective (1) delignification, (2) breaking lignin-carbohydrate bonds, (3) destroying the crystalline regions of cellulose, and (4) hydrolysis of polysaccharides. To achieve some or all of these goals, different approaches have been followed, which mostly operated at high temperatures for long durations (Table 2). The most common approach is achieving maximum wood disintegration in general without delignification. In such approach, the wood is dissolved completely then fully regenerated using water as antisolvent following the first fractionation strategy as shown in Figure 3 [110,117,118,119]. The disintegrated wood is then subjected to enzymatic hydrolysis using cellulase enzymes to produce glucose and other simple sugars, which are ultimately fermented to bioethanol. In some systems, the cellulase enzyme and ionic liquids were simultaneously used for wood disintegration and hydrolysis but they have to be compatible with each other in such systems [120,121,122]. Another approach disintegrates and delignifies wood and the dissolved lignin is separated using an acetone:water mixture or sodium hydroxide solution as antisolvent following the second fractionation strategy [112,123].

The most commonly used ionic liquids for wood disintegration were [EMIM][OAc], [AMIM][Cl], and [BMIM][Cl]. In general [EMIM][OAc] was more powerful than [AMIM][Cl], which was more powerful than [BMIM][Cl] [124]. In a few cases, DMSO was added to support further wood disintegration [125,126,127]. In some studies, aqueous solutions of ionic liquids were used to disintegrate wood by targeting the hemicelluloses as they are crucial for wood disintegration [58,128,129]. Similar studies aimed at selectively extracting the polysaccharides (mainly hemicelluloses) from wood to be hydrolyzed and fermented to biofuels at a later stage. In these studies, the acidic ionic liquid [BMIM][HSO_4_] was mainly used [96,130].

**Table 2:** Summary of the literature on wood and cellulose dissolution for biofuel production.

Wood/Lignocellulose	Dissolution Conditions			Regeneration		Ref.
	Ionic Liquid	Temp. (°C)	Time (h)	Strategy	Antisolvent	
Triticale, Wheat Straw	[EMIM][OAc]	150	1.5	2	0.1 M NaOH then Acidification	[112]
Cellulose	[EMIM][Cl], [AMIM][Cl], [BMIM][Cl]	100	2	1	Water	[131]
Radiata Pine, Eucalyptus	[EMIM][OAc]	50–150	0.8	1	Water	[132,133]
Scots Pine	[BHIM][HSO_4_] /Water	120–170	1–4	2	Ethanol then Water	[96]
Corn Stalk	[BMIM][BF_4_] /Water	150	5	1	Water	[117]
Yellow Pine	[EMIM][OAc]	140	0.25–0.75	1	Water	[134]
Guinea Grass	[EMIM][OAc]	157	0.5	1	Water	[118]
Japanese Cedar	[EMIM][OAc]	80	3	3	Acetone then Water	[110]
Japanese Cedar	[EMIM][OAc]	60–100	2–8	1	Water	[110]
Mixed Softwood	[BMIM][OAc]	100	15	1	Water	[119]
Hornbean, Spruce	[BMIM][Cl]	50–150	0.5–2	1	Water	[135]
Cedar, Eucalyptus, Bagasse Powder	[EMIM][OAc]	110	16	1	Water	[136]
Beech Wood Waste	[EMIM][OAc]	120	3	1	Water	[137]
Sugarcane	[BMIM][OAc]	110	0.5	3	Water	[138]
Poplar Wood Flour	[EMIM][OAc]	90	0.3–0.7	1	Water	[139]
Sawdust of Norway Spruce, Scots Pine, and Silver Birch and Winter Wheat Straw	[BMIM][OAc], [EMIM][Cl], [BMIM][Cl]	100–110	Up to 100	1	Water	[140]
Polar Wood	[BMIM][OAc], [MMIM][MEP]	130	18	2	Acetone then Water	[141]
Oil Palm Biomass	[EMIM][DEP]	70–100	4	2	1:1 Ac/W	[123]
Eastern White Pine	[AMIM][Cl] /DMSO	110	1	2	1:1 Ac/W	[125]
Norway Spruce	[BMIM][OAc]	100	20	1	Methanol	[142]
Pine Wood	[EMIM][Cl] /[EMIM][OAc] mixture	80–120	3	1	Water	[143]
Beech Wood	[EMIM][OAc] /Water	115	1.5	1	1:1 Ac/W	[81]
White Poplar and Pine	[EMIM][OAc] /DMSO	110	3	1	Water	[126]
P. tomentosa	[EMIM][OAc], [BMIM][Cl], [AMIM][Cl]	130	2	1	5% NaOH	[113]
Hybrid Pennisetum	[AMIM][Cl]	100–190	0.5–5.5	1	Water	[144]
Sugarcane Bagasse	[EMIM][OAc], [BMIM][Cl], [AMIM][Cl]	100	1	1	Water	[124]
Mixed Pine	[BMIM][Cl]	70–150	5–24	1	Water	[145]
Spruce and Oak Sawdust	[EMIM][OAc]	110	0.65	1	Methanol, Ethanol, Water	[146]
Silver Wattle	[EMIM][OAc]	90–150	0.5–24	1	Water, Ethanol, Methanol, 1:1 Ac/W	[147]
Sugarcane Bagasse Cellulose	[EMIM][OAc]	90	6	1	Water	[148]
Cassava Residues	[BMIM][Cl]	130	2	1	Water	[149]
Norway Spruce, Sugarcane	[AMIM][OF]	45–120	2–48	1	Water	[150]
Laminaria japonica Seaweed	[AMIM][Cl]	60–90	2	1	Water	[151]
Cotton Stalks	[EMIM][OAc]	150	0.5	1	Water	[152]
Spruce and Beech	[EMIM][OAc]	115	1.5	4	Ethanol then Water	[111]
Polar Wood	[EMIM][OAc]	110	12	1	Water	[153]
Birch and Pine Wood	[EMIM][OAc], [BMIM][Cl]	25	1–3	1	Ethanol	[154]
Aspen Wood	[EMIM][OAc]	120	1–5	1	Water	[155]
Eucalyptus	[EMIM][OAc] /DMSO	80–140	2–4	1	Water	[127]
Eucalyptus Cellulose	[EMIM][OAc], [AMIM][Cl], [BMIM][Cl], [BMIM][Ace]	130	1	1	Water	[94]
Douglas-Fir Wood	[EMIM][OAc]	120–160	3	1	Water	[156]
Yellow Pine	[HMIM][Cl]	110–150	Up to 5	2	1:1 Ac/W	[54]
Pine, Eucalyptus, Switchgrass	[EMIM][OAc]	160	3	1	Water	[157]
Norway Spruce	[EMIM][OAc], [BMIM][OAc]	120	1–15	1	Water	[158]
Sago Waste	[BMIM][Cl]	140–155	0.5–2.5	1	Water	[159]
Oil Palm Fronds	[BMIM][Cl]	80–120	0.5–3	1	Water	[160]
Kenaf Powder	[BMIM][Cl], [AMIM][Cl], [EMIM][Cl], [EMIM][DEP], [EMIM][OAc]	110	2	1	Water	[161]
Sugarcane Bagasse	[EMIM][OAc]	120	2	1	Water	[162]
Sugarcane Bagasse	[BMIM][Cl]	150	1.5	1	Water	[163]
Wheat Straw	[EMIM][OAc] /Water	130–170	0.5–5.5	1	Water	[128,129]
Miscanthus giganteus, Pine, Willow	[BMIM][HSO_4_] /Water, [BMIM][MSO_4_] /Water	120	2	3	Water	[130]
Eucalyptus, Southern Pine, Norway spruce	[AMIM][Cl]	120	5	1	Water, Methanol	[106]
Switch grass	[BMIM][Cl], [EMIM][OAc], [BMIM][OAc]	110	0.25	1	Water	[59]
Maple Wood Flour	[EMIM][OAc]	50–130	0.5–20	1	Water	[57]

Abbreviations: [OF]: formate, [DEP]: diethylphosphate, [MMIM]: 1-methyl-3-methylimidazolium, [MEP]: methylphosphite, [MSO_4_]: methylsulfate, Ac/W: acetone/water mixture.

### 4.2. Ionic Liquid-Mediated Fractionation and Pulping of Wood and Lignocellulose

Traditional wood pulping, including Kraft and Sulfite pulping, are considered environmentally unfriendly due to the use of strong acidic and alkaline solutions, which require special treatments [164,165]. Furthermore, the separation of dissolved hemicelluloses and lignin from the pulping liquor is relatively difficult. When extracted, lignin and hemicelluloses can be strongly modified (sulfonation, oxidation) during pulping limiting their potential in certain applications [166]. Ionic liquids offer a solution for these challenges and provide many possibilities for wood fractionation and pulping, depending on the aim of the treatment. The fractionation procedure is also relatively simple, and the separated fractions are not/slightly modified [167]. 

Some fractionation procedures dissolve wood completely and fraction it to its individual components without paying attention to the crystalline structure of cellulose (Table 3) [168,169]. The individual polymers are then used separately or together for certain applications. For example, cellulose and hemicelluloses can be used for the production of bioethanol, furfural compounds [170], pyrolysis products [108], and synthetic wood composites [171] while lignin can be used for the production of adhesives and phenolic compounds [172,173]. Sometimes, wood components are selectively extracted. Acesulfamate and methylsulfate ionic liquids were suggested as selective solvents for lignin [50,174], while a mixture of ionic liquids with water and certain organic solvents were proved efficient for the extraction of hemicelluloses [175]. The aqueous and organic mixture of ionic liquids were used to upgrade Kraft pulp to high-quality dissolving pulp by reducing its hemicellulose content [176,177,178]. Ionic liquids acidified to a pH of 4–5 were found to be able to dissolve hemicelluloses selectively. If the pH is adjusted to 2–3, they become capable of dissolving cellulose. In both scenarios, lignin stays undissolved [87]. During the selective extraction of hemicelluloses and lignin, the crystalline structure could be degraded, especially if high dissolution temperatures are used. At lower temperatures (60 °C and less) and short dissolution time (two hours and less), it was possible to extract Cellulose I pulp [179,180]. Different studies have shown that the dissolution of hemicelluloses and cellulose is mainly affected by the anion, while lignin dissolution is affected by both the cation and anion [181,182,183]. Significant pi-pi stacking of imidazolium with the benzene rings supports lignin dissolution and depolymerization [184,185].

### 4.3. Ionic Liquids for Processing All-Wood and All-Cellulose Composites

Wood liquefaction is necessary for its processing and fractionation. In the past, it was not possible to liquefy wood without the use of high pressure and temperature. Various liquefaction and dissolution agents were then explored, such as phenol and ethylene carbonate, in the presence of strong acids as catalysts [191,192]. In 2006, Honglu and Teijun showed that ionic liquids, in addition to their green properties, can be better wood liquefaction agents than phenol/sulfuric acid [26]. Since then, ionic liquids were explored as solvents and liquefaction agents for wood modification [193], wood processing [194], and to facilitate wood grinding [195]. They were also used for processing synthetic wood composites of controlled amounts of cellulose/hemicelluloses/lignin [171].

Wood liquefaction with ionic liquids causes major changes in the anatomic and molecular levels, including swelling, fibrillation, disintegration, plasticization, and derivatization [66,196]. Wood swelling in ionic liquids is comparable, and sometimes better, to that in water and organic solvents [66]. It is also reversible, which means that cellulose can keep its native crystalline structure upon the removal of the ionic liquid [197], although strong fibrillation and disintegration are observed. Thermomechanical studies showed that ionic liquids are capable of lowering the glass transition of lignin in wood to values similar to or lower than those obtained using water as a plasticizer [198]. [EMIM][OAc]-plasticized Norway spruce showed a glass transition around 60 °C, which is lower than those obtained using water and ethylene glycol (84 and 74 °C, respectively) [199,200]. Such kind of strong plasticization and swelling are advantageous for wood processing, as they determine its energy requirement [199,201,202].

All-wood and all-cellulose composites have been processed following one main procedure (Table 4). The wood or cellulose is partially or completely dissolved in an ionic liquid before hot-pressed [203]. The ionic liquid is taken out of the composite, and the wood is fully regenerated before or after pressing by washing or extraction using an antisolvent such as water or ethanol. Sometimes, the composite is made of a mixture of wood and cellulose [204,205,206]. Other times, the plasticized wood is processed with another polymer such as thermoplastic starch [207].

### 4.4. Extraction of Cellulose Nanoparticles from Wood and Cellulose

Upon wood pulping, cellulose is extracted in the form of 20–40 µm-thick fibers, which can be processed to nanocellulose using a wide range of chemical and mechanical procedures [45]. Nanocellulose is an interesting material because of its high mechanical strength, high surface area, the ability to modify its surface and to form liquid crystalline structures, in addition to biodegradability and biocompatibility [214,215,216,217]. Thanks to these properties, nanocellulose has shown great potential in a wide range of applications, including automotive industry [218], water filtration [219,220], tissue engineering [221], pharmaceutical formulation [222], electronics [223], etc. Cellulose nanocrystals (CNCs), one form of nanocellulose, are rod-like nanoparticles with a thickness of 3–10nm and a length of a few hundreds of nanometers [224]. They are extracted chemically by hydrolyzing the amorphous regions of cellulose using strong acids using a procedure that has already been industrialized [45]. Sulfuric acid is the most commonly used for the extraction of CNCs, although many such as hydrochloric acid, phosphoric acid, and acetic acid have been also explored [45]. The acid-based procedure suffers high severity, corrosion of reactors, the need to treat the effluents, and the low CNC yield, which is in the range of 20–40% (g-CNCs/g-cellulose) [225,226,227]. 

Ionic liquids have been explored to extract CNCs under milder, controllable, and greener conditions. Most of the reports in the literature focused on the use of the mildly acidic ionic liquid, 1-butyl-3-methylimidazolium hydrogen sulfate [BMIM][HSO_4_] [228]. Man et al. have used this ionic liquid for the first time in 2011 to extract CNCs, but the extracted cellulose did not have the optimum morphological characteristics of CNCs [229]. Mao et al. were able to extract higher quality CNCs in almost theoretical yields using the same ionic liquid upon dilution with water [44,230,231]. Grzabka-Zasadzinska et al. studied the impact of the imidazolium substituents on the properties of the CNCs extracted using this ionic liquid [232]. Regenerated CNCs were also produced upon the dissolution of cellulose in [BMIM][Cl] [233].

Cellulose nanofibrils (CNFs), another form of nanocellulose, are spaghetti-like nanoparticles with a thickness of 5–30 nm and a length of few micrometers [224]. They are produced by mechanically fibrillating cellulose fibers using a wide range of techniques, including homogenization, microfluidization, microgrinding, and extrusion [234,235,236,237]. There are some reports that used ionic liquids to prepare regenerated CNFs [238]. In these reports, cellulose is dissolved in an ionic liquid then electrospun. The produced CNFs are significantly thicker than traditional CNFs (thickness of around 500 nm) [239]. Mao et al. have reported the use of imidazole to produce both CNCs and CNFs. Imidazole, alone, was found able to fibrillate cellulose to produce CNFs, and to hydrolyze the amorphous regions of cellulose to liberate the CNCs when mixed with water [240].

Most of the reports in the literature on the production of CNCs and CNFs used pure or almost pure cellulose as a starting material, including pulp fibers and microcrystalline cellulose [45]. In other words, there were no technologies for the extraction of nanocellulose directly from wood and lignocelluloses. This is not surprising, as there was no single reagent that is capable of pulping wood and extracting nanocellulose at the same time. Using [EMIM][OAc], Abushammala et al. developed a method to pulp wood and simultaneously hydrolyze the amorphous regions of the cellulose to liberate the crystallite in the form of CNCs [180,241]. The extracted CNCs were lignin-coated, which can be bleached to obtain lignin-free CNCs [179]. Such technology could foster the utilization of lignocellulosic residues and the use of naoncellulose [242]. Ionic liquids were also used for the preparation of lignin nano- and micro-spheres by dissolving alkali lignin in [MMIM][DEP] followed by a gradual regeneration in water [243].

## 5. Challenges of Using Ionic Liquids for Wood and Cellulose Dissolution and Processing

Around 20 years ago, ionic liquids were introduced as the “magic solvents” for processing wood and cellulose and received strong attention due to their advantageous properties. Years later, it became clear that ionic liquids are not that innocent, as they seemed to be. Many issues and challenges appeared that hindered industrializing some promising ionic liquid-mediated technologies developed in academia for processing wood and cellulose:

### 5.1. Side Reactions

For many years, it was assumed that ionic liquids are chemically inert solvents for cellulose and wood. However, recent studies have reported possible side reactions that could take place during processing cellulose and wood using ionic liquids. Acetylation has been reported to take place at varying degrees using acetate ionic liquids, such as [EMIM][OAc]. Small degrees of cellulose acetylation (less than 0.1%) were reported when cellulose was treated with this ionic liquid [244,245]. Acetylation was proven to be caused by one of the impurities in the ionic liquid, 1-acetylimidazole [246]. This impurity is continuously generated because of the thermal degradation of the ionic liquid during the reaction and during the recycling of the ionic liquid. Acetylation was more significant (>10%) in the presence of certain molecules such as 2-furoyl chloride, which provided an electropositive carbon for the acetate ion to attach and be activated [247]. Cellulose acetylation was also more significant when wood was treated with [EMIM][Ace] (>10%) [180]. It was confirmed, in this scenario, that lignin acted as a catalyst [248].

Many studies have also reported the hydrolysis of cellulose when treated with ionic liquids [179,180,249,250]. Some attributed it to the formation of acetic acid, when acetate ionic liquids are used or because of hemicellulose deacetylation [248,251,252], while others linked it to the hydrolytic power of the imidazolium ring [253]. The hydrolytic power of imidazolium is a result of releasing the proton of the ring by the action of the anion [254,255]. This was more significant for acetate ionic liquids compared to chloride ionic liquids due to the high basicity of the acetate ion [256]. In the process, a carbene is formed, which is reactive with the reducing end and C2 of cellulose [254,257,258,259]. This explains the traces of nitrogen detected in cellulose after processing with [EMIM][OAc]. Other than the degradation of the cation, the anions can also degrade. For instance, the acesulfamate anion was proved to degrade significantly to ammonium sulfate salt, which was detected in significant amounts in lignin extracted from wood using acesulfamate ionic liquids [260].

Sulfonation and phosphorylation were also detected when wood and cellulose were processed with [BMIM][HSO_4_] and [EMIM][MEP], respectively [141,230]. Acidic ionic liquids such as [EMIM][HSO_4_] and [BMIM][HSO_4_] were found to convert sugars to 5-hydroxymethylfufural [261,262]. Sometimes, these side reactions, in addition to acetylation and hydrolysis, were advantageous as they supported the dissolution of lignin and cellulose [141]. For instance, the acetylation of lignin and cellulose and hydrolysis of cellulose during wood treatment with [EMIM][OAc] supported the direct extraction of CNCs from wood through simultaneous wood delignification, cellulose fibrillation, and liberation of cellulose crystallites. Lignin acetylation prevented lignin from repolymerizing while cellulose acetylation reduced the cohesive forces between cellulose microfibrils during pulping, preventing them from aggregating [248].

### 5.2. Recyclability

Most of the reports in the literature recycled ionic liquids upon wood dissolution or processing by evaporating all added co-solvents and anti-solvents after the separation of all wood fractions relying on the fact that ionic liquids will not evaporate at the typical drying temperatures [114]. It was often claimed that ionic liquids are recyclable with almost no losses due to their low vapor pressure and high thermal stability. However, many studies have shown that ionic liquids degrade due to the high temperatures used for wood and cellulose dissolution [263]. Their degradation might also be catalyzed by the wood moieties generated during dissolution [260]. The side reactions mentioned previously are also a possible cause of losses [180,247].

Ionic liquid recyclability is not only assessed by mass loss but also by the performance of the ionic liquid after recycling. Under high dissolution temperatures, wood could degrade, and oligomers could form, which cannot be regenerated out of the ionic liquid. These oligomers accumulate in the ionic liquid reducing its efficiency [264,265]. The accumulation of wood oligomers, in addition to thermal degradants, can be visually detected as ionic liquids tend to darken [230]. It is worth to mention that it could be possible to evaporate ionic liquids to get rid of these oligomers. Such a process would need a high-energy input in the form of heat and vacuum [266,267]. Some approaches have been developed to overcome this issue, including the formation of distillable carbenes and the back-alkylation of the anion [268]. In conclusion, lowering wood dissolution temperature, if possible, is recommended to suppress both wood and ionic liquid degradation.

### 5.3. High Viscosity and Low Wood/Ionic Liquid Mixing Ratio

Some of the commonly used ionic liquids are solid at room temperature, while others have a viscosity of 100-2000 cP at room temperature (Table 1). Halide- and hydrogen sulfate-based ionic liquids tend to be solid or have a high viscosity of more than 1000 CP while acetate, tetrafluoroborate, and hexafluorophosphate ionic liquids are liquid and have a viscosity of few hundreds’ cP or less. These viscosities are still significantly higher than the viscosities of commonly used organic solvents (less than 2 cP) [269]. The high viscosity of ionic liquids has a negative impact on four major process parameters: (1) energy input (2) solid/liquid ratio, (3) reaction homogeneity, and (4) filtration of the reaction mixture. High temperature is needed to melt and reduce the viscosity of ionic liquid, while vigorous mixing is needed to improve reaction homogeneity. To improve the solid/liquid ratio and allow the filtration of the reaction mixture, cosolvents such as DMSO are added to the ionic liquid to reduce their viscosity, which would result in an extra effort in ionic liquid recycling at the end [188].

### 5.4. Prices of Ionic Liquids

The prices of most imidazolium ionic liquids ranged in 2019 between 500 and 5000 euros per kilogram (Table 5) [270,271,272,273]. These prices are up to 100 times higher than commonly used organic solvents such as acetone, ethanol, toluene, and DMSO [270,271,272,273], which consequently hinder the inclusion of ionic liquids in many applications and processes. However, these prices are lower than those in the past and will continue to drop due to the growth of the ionic liquids market [22].

### 5.5. Health and Environmental Concerns

The possibility to synthesize a huge number of ionic liquids with tuned properties fostered their use in a wide range of applications. However, this requires thorough studies of the health and environmental impact of all newly synthesized ionic liquids before commercialization. This is important due to the fact that ionic liquids are not innocent solvents as they seemed to be, and some traces may exist in final products even upon careful washing and as a result of side reactions. They may also leash to water and soil [274,275]. Some toxicity studies have shown that ionic liquids are/could be toxic to humans and animals [276,277,278,279,280], and microorganisms [281,282,283], which was mainly linked to the imidazolium cation and influenced by the anion [280,284,285]. Other studies have shown that using ionic liquid for cellulose dissolution might be less environmentally friendly than the traditional NMMO process [286]. Due to the toxicity of the imidazolium ring, other cations have been suggested, such as morpholinium and choline [287,288], which showed similar and sometimes better performance as solvents [289]. Amino acids as anions have been also explored [290,291]. In conclusion, there is a strong need to standardize a system to study the impact of ionic liquids on human health (including genotoxicity, cytotoxicity, and neurotoxicity) and to understand their fate and transport in the environment. A systematic approach to tackle this issue would further accelerate the inclusion of ionic liquids in chemical processes and commercial products.

## 6. Conclusions

Ionic liquids offer a variety of possibilities for the dissolution, fractionation, and processing of wood, lignocelluloses, and cellulose. They allow the utilization of these bioresources for biofuel production, processing wood-based composites, extraction of nanocellulose, and the production of a variety of chemicals and materials. This is a result of the possibility to synthesize unlimited number of ionic liquids with tailored chemical and physical properties and controlled dissolution power and selectivity to wood and its polymers. Ionic liquids have been able to destruct wood and significantly facilitate the enzymatic hydrolysis of its polysaccharides. They have also been proven potent agents for wood pulping and fractionation and for the extraction of cellulose nanoparticles from cellulose and wood. For processing all-wood composites, they have shown superior plasticization capabilities compared to water and traditional plasticization agents. Despite their fascinating performance, ionic liquids are not innocent and green solvents as they seemed to be two decades ago. Many side reactions have been reported, including acetylation, hydrolysis, and thermal and chemical degradation, which significantly affect their recyclability. They also suffer high viscosity and are still more expensive than commonly used organic solvents. However, their market continues to grow due to their inclusion in more applications and processes.

## Figures and Tables

**Table 3:** Summary of the literature on wood fractionation for the separation of its individual polymers.

Wood/Lignocellulose	Dissolution Conditions			Regeneration		Ref.
	Ionic Liquid	Temp. (°C)	Time (h)	Strategy	Antisolvent	
Cypress Wood	[EMIM][OAc]	80	1	2	1:1 Ac/W	[186]
Rubber Wood	[MMIM][MSO_4_]	25–100	0.5–2.5	3	Methanol	[187]
Sugarcane Straw	[EMIM][OAc]	90	5	2	1:1 Ac/W	[108]
Eucalyptus urophylla	[EMIM][OAc]	140–170	0.5–6	4	Water then Acidic Water	[97]
Maritime Pine	[BMIM][HSO_4_] /Water	170	0.5–2	2	Ethanol then Water	[170]
Norway spruce	[EMIM][OAc] /DMSO	80–150	2–6	4	Acetone	[188]
Steam-exploded Angelim Vermelho	[EMIM][OAc]	30	0.25	4	1:1 Ac/W	[179]
Angelim Vermelho	[EMIM][OAc]	60	2	4	1:1 Ac/W	[180]
Wheat Straw	[EMIM][OAc]	110–120	1–16	1	0.1M NaOH or 9:1 Ac/W	[109]
Radiata Pine, Eucalyptus globlus	[AMIM][Cl]	120–170	0.3–1	4	DMSO then Methanol	[102,103]
Birch Wood	[EMIM][OAc]	110	16	2	1:1 Ac/W	[189]
Wheat Straw	[EMIM][OAc]	80–140	2–18	2	Alkaline Water, Neutral, then Acidic	[168]
Poplar Wood	[EMIM][OAc]	110	16	2	1:1 Ac/W	[167]
Sugarcane Bagasse	[BMIM][Cl]	110	72	2	9:1 Ac/W	[169]
Bagasse	[EMIM][Xylenesulfonate]	170–190	0.5–2	1	0.1M NaOH then Acidification	[190]
Southern Pine, Red Oak	[EMIM][OAc], [BMIM][OAc]	110	16	2	1:1 Ac/W	[61]
Radiata Pine	[BMIM][Ace]	80–140	1–16	3	Acetone	[50]

**Table 4:** Summary of the literature on processing all-wood and all-cellulose composites using ionic liquids.

Wood/Lignocellulose	Dissolution Conditions			Regeneration Mechanism		Ref.
	Ionic Liquid	Temp. (°C)	Time (h)	Strategy	Antisolvent	
Wheat Straw and Cellulose	[AMIM][Cl]	120	4	1	Casting then Water	[208]
Chinese Fir	[AMIM][Cl]	80	4	1	Water then Hot-pressing	[203]
Birch Wood	[EMIM][OAc]	95	0.5	1	Water and Hot-pressing	[209]
Paper Cellulose	[EMIM][OAc] /Water	80–95	1–16	1	Water then Wet-pressing and Drying	[210]
Oil Palm Frond	[EMIM][OAc], [BMIM][Cl]	90	3	1	1:1 Ac/W then Hot-pressing with Starch	[207]
Bleached and Unbleached Soda Pulps	[BMIM][Cl]	85–95	1	1	Casting then Water	[211]
Poplar Wood with Paper	[EMIM][OAc]	100	1	1	Hot-pressing then Water	[205]
Lyocell Fibers	[BMIM][Cl]	110	0.5–4	1	Hot-pressing then Water	[204]
Aspen Wood with Cotton	[EMIM][OAc]	60–80	0.5–20	1	Water then Hot-pressing	[212]
Cotton, Japanese Cypress Lumber	[BMIM][Cl]	100	0.5	1	Hot-pressing then Acetonitrile	[206]
Cedar Flour, Bark Flour	[BMIM][Cl]	100	0.15	1	Hot-pressing then Ethanol	[213]
Cedar, Bark Flour	[BMIM][Cl]	100	0.2	1	Extraction with Ethanol	[213]

**Table 5:** The prices of the most commonly used ionic liquids for wood and cellulose processing. The prices are not enlisted for comparison as they are based on products of different purities (95–100%) and sizes (5–1000 g).

Ionic Liquid	Price (EUR/kg)			
	Sigma-Aldrich [270]	TCI [271]	Alfa Aesar [272]	Acros [273]
[BMIM][OAc]	821	-	-	-
[BMIM][Cl]	1264	1250	2300	882
[BMIM][Br]	517	5600	1836	-
[BMIM][I]	4880	5200	-	-
[BMIM][HSO_4_]	421	3080	-	-
[BMIM][BF_4_]	1776	1420	5940	2070
[BMIM][PF_6_]	1992	2520	2440	2390
[EMIM][OAc]	746	7160	15,580	-
[EMIM][Cl]	363	1224	4680	1080
[EMIM][Br]	2080	3520	1800	1250
[EMIM][I]	3960	3400	3720	-
[EMIM][HSO_4_]	511	2880	1572	-
[EMIM][BF_4_]	5660	6000	-	-
[EMIM][PF_6_]	9000	5040	6380	-
[AMIM][Cl]	5240	6000	4880	-
[AMIM][Br]	21,600	-	2940	-
[AMIM][I]	14,400	-	-	-
[BnMIM][Cl]	4600	7000	4880	-
[BnMIM][BF_4_]	4840	4800	5580	-
[BnMIM][PF_6_]	4740	6200	4960	-

## References

[1] Sivasubramanian V. (2018). Bioprocess Engineering for a Green Environment.

[2] Tumuluru J.S. (2018). Biomass Preprocessing and Pretreatments for Production of Biofuels: Mechanical, Chemical and Thermal Methods.

[3] Sun R. (2010). Cereal Straw as a Resource for Sustainable Biomaterials and Biofuels: Chemistry, Extractives, Lignins, Hemicelluloses and Cellulose.

[4] Klemm D., Heublein B., Fink H.P., Bohn A. (2005). Cellulose: Fascinating biopolymer and sustainable raw material. Angew. Chem. Int. Ed..

[5] Vishtal A.G., Kraslawski A. (2011). Challenges in industrial applications of technical lignins. BioResources.

[6] Searle S., Malins C. (2013). Availability of Cellulosic Residues and Wastes in the EU.

[7] Council E.P. (2018). Bioenergy Europe Pellet Report.

[8] Parthasarathi R., Bellesia G., Chundawat S., Dale B., Langan P., Gnanakaran S. (2011). Insights into hydrogen bonding and stacking interactions in cellulose. J. Phys. Chem. A.

[9] Himmel M.E., Ding S.-Y., Johnson D.K., Adney W.S., Nimlos M.R., Brady J.W., Foust T.D. (2007). Biomass recalcitrance: Engineering plants and enzymes for biofuels production. Science.

[10] Williamson S.L., McCormick C.L. (1998). Cellulose derivatives synthesized via isocyanate and activated ester pathways in homogeneous solutions of lithium chloride/N, N-dimethylacetamide. J. Macromol. Sci. Pure Appl. Chem..

[11] Rosenau T., Potthast A., Sixta H., Kosma P. (2001). The chemistry of side reactions and byproduct formation in the system NMMO/cellulose (Lyocell process). Prog. Polym. Sci..

[12] Welton T. (1999). Room-temperature ionic liquids. Solvents for synthesis and catalysis. Chem. Rev..

[13] Wang H., Gurau G., Rogers R.D. (2012). Ionic liquid processing of cellulose. Chem. Soc. Rev..

[14] Huddleston J.G., Visser A.E., Reichert W.M., Willauer H.D., Broker G.A., Rogers R.D. (2001). Characterization and comparison of hydrophilic and hydrophobic room temperature ionic liquids incorporating the imidazolium cation. Green Chem..

[15] Rogers R.D., Seddon K.R. (2003). Ionic liquids--solvents of the future?. Science.

[16] Lewandowski A., Świderska-Mocek A. (2009). Ionic liquids as electrolytes for Li-ion batteries—An overview of electrochemical studies. J. Power Sources.

[17] De Souza R.F., Padilha J.C., Gonçalves R.S., Dupont J. (2003). Room temperature dialkylimidazolium ionic liquid-based fuel cells. Electrochem. Commun..

[18] Dupont J., de Souza R.F., Suarez P.A. (2002). Ionic liquid (molten salt) phase organometallic catalysis. Chem. Rev..

[19] Huang K., Chen F.-F., Tao D.-J., Dai S. (2017). Ionic liquid–formulated hybrid solvents for CO_2_ capture. Curr. Opin. Green Sustain. Chem..

[20] Meindersma G.W., De Haan A.B. (2012). Cyano-containing ionic liquids for the extraction of aromatic hydrocarbons from an aromatic/aliphatic mixture. Sci. China Chem..

[21] Angell C.A., Ansari Y., Zhao Z. (2012). Ionic liquids: Past, present and future. Faraday Discuss..

[22] Kunal Ahuja S.D. (2016). Ionic Liquids Market Size by Application (Catalysis/Synthesis, Food, Paper & Pulp, Electronics, Biotechnology, Automotive, Pharmaceuticals), Industry Analysis Report, Regional Outlook, Application Potential, Price Trends, Competitive Market Share & Forecast, 2015–2022.

[23] Swatloski R.P., Spear S.K., Holbrey J.D., Rogers R.D. (2002). Dissolution of cellose with ionic liquids. J. Am. Chem. Soc..

[24] Charles G. (1933). Cellulose Solution and Cellulose Derivative and Process of Making Same. U.S. Patents.

[25] Charles G. (1934). Cellulose Solution. U.S. Patents.

[26] Honglu X., Tiejun S. (2006). Wood liquefaction by ionic liquids. Holzforschung.

[27] Li X., Geng Y., Simonsen J., Li K. (2004). Application of ionic liquids for electrostatic control in wood. Holzforschung.

[28] Pernak J., Zabielska-Matejuk J., Kropacz A., Foksowicz-Flaczyk J. (2004). Ionic liquids in wood preservation. Holzforschung.

[29] Pernak J., Goc I., Fojutowski A. (2005). Protic ionic liquids with organic anion as wood preservative. Holzforschung.

[30] Kilpeläinen I., Xie H., King A., Granstrom M., Heikkinen S., Argyropoulos D.S. (2007). Dissolution of wood in ionic liquids. J. Agric. Food Chem..

[31] Fort D.A., Remsing R.C., Swatloski R.P., Moyna P., Moyna G., Rogers R.D. (2007). Can ionic liquids dissolve wood? Processing and analysis of lignocellulosic materials with 1-n-butyl-3-methylimidazolium chloride. Green Chem..

[32] Seddon K.R. (1997). Ionic liquids for clean technology. J. Chem. Technol. Biotechnol. Int. Res. Process Environ. AND Clean Technol..

[33] Pinkert A., Marsh K.N., Pang S., Staiger M.P. (2009). Ionic liquids and their interaction with cellulose. Chem. Rev..

[34] Wasserscheid P., Welton T. (2008). Ionic liquids in synthesis.

[35] MacFarlane D.R., Forsyth S.A. (2003). Acids and bases in ionic liquids.

[36] Plechkova N.V., Seddon K.R. (2008). Applications of ionic liquids in the chemical industry. Chem. Soc. Rev..

[37] Rooney D., Jacquemin J., Gardas R. (2009). Thermophysical properties of ionic liquids. Ionic Liquids.

[38] Handy S.T. (2005). Room temperature ionic liquids: Different classes and physical properties. Curr. Org. Chem..

[39] Xu A., Zhang Y., Li Z., Wang J. (2012). Viscosities and conductivities of 1-butyl-3-methylimidazolium carboxylates ionic liquids at different temperatures. J. Chem. Eng. Data.

[40] Meindersma G.W., Maase M., De Haan A.B. (2000). Ionic liquids. Ullmann’s Encyclopedia of Industrial Chemistry.

[41] Othman Z.S., Hassan N.H., Zubairi S.I. (2017). Imidazolium-Based Ionic Liquid Binary Solvent System as an Extraction Medium in Enhancing the Rotenone Yield Extracted from Derris elliptica Roots. Progress and Developments in Ionic Liquids.

[42] Chancelier L., Boyron O., Gutel T., Santini C. (2016). Thermal stability of imidazolium-based ionic liquids. Fr. Ukr. J. Chem..

[43] Erdmenger T., Vitz J., Wiesbrock F., Schubert U.S. (2008). Influence of different branched alkyl side chains on the properties of imidazolium-based ionic liquids. J. Mater. Chem..

[44] Mao J., Heck B., Reiter G., Laborie M.-P. (2015). Cellulose nanocrystals’ production in near theoretical yields by 1-butyl-3-methylimidazolium hydrogen sulfate ([Bmim] HSO4)–mediated hydrolysis. Carbohydr. Polym..

[45] Mao J., Abushammala H., Brown N., Laborie M.-P. (2017). Comparative assessment of methods for producing cellulose I nanocrystals from cellulosic sources. Nanocelluloses: Their Preparation, Properties, and Applications, ACS Symposium Series.

[46] Liu C., Li Y., Hou Y. (2018). Basicity characterization of imidazolyl ionic liquids and their application for biomass dissolution. Int. J. Chem. Eng..

[47] Zhang S., Lu X., Zhou Q., Li X., Zhang X., Li S. (2009). Ionic Liquids: Physicochemical Properties.

[48] Iolitec Website. www.iolitec.de.

[49] Han D., Row K.H. (2010). Recent applications of ionic liquids in separation technology. Molecules.

[50] Pinkert A., Goeke D.F., Marsh K.N., Pang S. (2011). Extracting wood lignin without dissolving or degrading cellulose: Investigations on the use of food additive-derived ionic liquids. Green Chem..

[51] Feng L., Chen Z.-L. (2008). Research progress on dissolution and functional modification of cellulose in ionic liquids. J. Mol. Liq..

[52] Isik M., Sardon H., Mecerreyes D. (2014). Ionic liquids and cellulose: Dissolution, chemical modification and preparation of new cellulosic materials. Int. J. Mol. Sci..

[53] Zavrel M., Bross D., Funke M., Büchs J., Spiess A.C. (2009). High-throughput screening for ionic liquids dissolving (ligno-) cellulose. Bioresour. Technol..

[54] Cox B.J., Ekerdt J.G. (2013). Pretreatment of yellow pine in an acidic ionic liquid: Extraction of hemicellulose and lignin to facilitate enzymatic digestion. Bioresour. Technol..

[55] Pang J., Liu X., Zhang X., Wu Y., Sun R. (2013). Fabrication of cellulose film with enhanced mechanical properties in ionic liquid 1-allyl-3-methylimidaxolium chloride (AmimCl). Materials.

[56] Jiang M., Zhao M., Zhou Z., Huang T., Chen X., Wang Y. (2011). Isolation of cellulose with ionic liquid from steam exploded rice straw. Ind. Crop. Prod..

[57] Lee S.H., Doherty T.V., Linhardt R.J., Dordick J.S. (2009). Ionic liquid-mediated selective extraction of lignin from wood leading to enhanced enzymatic cellulose hydrolysis. Biotechnol. Bioeng..

[58] Moyer P., Smith M.D., Abdoulmoumine N., Chmely S.C., Smith J.C., Petridis L., Labbé N. (2018). Relationship between lignocellulosic biomass dissolution and physicochemical properties of ionic liquids composed of 3-methylimidazolium cations and carboxylate anions. Phys. Chem. Chem. Phys..

[59] Zhao H., Baker G.A., Cowins J.V. (2010). Fast enzymatic saccharification of switchgrass after pretreatment with ionic liquids. Biotechnol. Prog..

[60] Barthel S., Heinze T. (2006). Acylation and carbanilation of cellulose in ionic liquids. Green Chem..

[61] Sun N., Rahman M., Qin Y., Maxim M.L., Rodríguez H., Rogers R.D. (2009). Complete dissolution and partial delignification of wood in the ionic liquid 1-ethyl-3-methylimidazolium acetate. Green Chem..

[62] Brandt A., Hallett J.P., Leak D.J., Murphy R.J., Welton T. (2010). The effect of the ionic liquid anion in the pretreatment of pine wood chips. Green Chem..

[63] Vo H.T., Kim C.S., Ahn B.S., Kim H.S., Lee H. (2011). Study on dissolution and regeneration of poplar wood in imidazolium-based ionic liquids. J. Wood Chem. Technol..

[64] Strehmel V., Strunk D., Wetzel H., Strehmel N. (2017). Investigation of lignin obtained by processing of Betula pendula with ionic liquids. Sustain. Chem. Pharm..

[65] Yang Y., Fan H., Meng Q., Zhang Z., Yang G., Han B. (2017). Ionic liquid [OMIm][OAc] directly inducing oxidation cleavage of the β-O-4 bond of lignin model compounds. Chem. Commun..

[66] Abushammala H., Pontes J.F., Gomes G.H., Osorio-Madrazo A., Thiré R.M., Pereira F.V., Laborie M.-P.G. (2015). Swelling, viscoelastic, and anatomical studies on ionic liquid-swollen Norway spruce as a screening tool toward ionosolv pulping. Holzforschung.

[67] Wang Z., Gräsvik J., Jönsson L.J., Winestrand S. (2017). Comparison of [HSO_4_]^−^,[Cl]^−^ and [MeCO_2_]^−^ as anions in pretreatment of aspen and spruce with imidazolium-based ionic liquids. BMC Biotechnol..

[68] De Gregorio G.F., Weber C.C., Gräsvik J., Welton T., Brandt A., Hallett J.P. (2016). Mechanistic insights into lignin depolymerisation in acidic ionic liquids. Green Chem..

[69] Padró J.M., Reta M. (2016). Solvatochromic parameters of imidazolium-, hydroxyammonium-, pyridinium- and phosphonium-based room temperature ionic liquids. J. Mol. Liq..

[70] Doherty T.V., Mora-Pale M., Foley S.E., Linhardt R.J., Dordick J.S. (2010). Ionic liquid solvent properties as predictors of lignocellulose pretreatment efficacy. Green Chem..

[71] Liu R., Zhang J., Sun S., Bian Y., Hu Y. (2019). Dissolution and recovery of cellulose from pine wood bits in ionic liquids and a co-solvent component mixed system. J. Eng. Fibers Fabr..

[72] Fendt S., Padmanabhan S., Blanch H.W., Prausnitz J.M. (2010). Viscosities of acetate or chloride-based ionic liquids and some of their mixtures with water or other common solvents. J. Chem. Eng. Data.

[73] Lee Y.J., Kwon M.K., Lee S.J., Jeong S.W., Kim H.C., Oh T.H., Lee S.G. (2017). Influence of water on phase transition and rheological behavior of cellulose/ionic liquid/water ternary systems. J. Appl. Polym. Sci..

[74] Ding Z.-D., Chi Z., Gu W.-X., Gu S.-M., Liu J.-H., Wang H.-J. (2012). Theoretical and experimental investigation on dissolution and regeneration of cellulose in ionic liquid. Carbohydr. Polym..

[75] Marks C., Mitsos A., Viell J. (2019). Change of C (2)-Hydrogen–Deuterium Exchange in Mixtures of EMIMAc. J. Solut. Chem..

[76] Castro M.C., Arce A., Soto A., Rodríguez H.c. (2015). Influence of methanol on the dissolution of lignocellulose biopolymers with the ionic liquid 1-ethyl-3-methylimidazolium acetate. Ind. Eng. Chem. Res..

[77] Sun Y., Xue B. (2018). Understanding structural changes in the lignin of Eucalyptus urophylla during pretreatment with an ionic liquid-water mixture. Ind. Crop. Prod..

[78] Laine C., Asikainen S., Talja R., Stépán A., Sixta H., Harlin A. (2016). Simultaneous bench scale production of dissolving grade pulp and valuable hemicelluloses from softwood kraft pulp by ionic liquid extraction. Carbohydr. Polym..

[79] Shi J., Balamurugan K., Parthasarathi R., Sathitsuksanoh N., Zhang S., Stavila V., Subramanian V., Simmons B.A., Singh S. (2014). Understanding the role of water during ionic liquid pretreatment of lignocellulose: Co-solvent or anti-solvent?. Green Chem..

[80] Roselli A., Hummel M., Vartiainen J., Nieminen K., Sixta H. (2017). Understanding the role of water in the interaction of ionic liquids with wood polymers. Carbohydr. Polym..

[81] Viell J., Inouye H., Szekely N.K., Frielinghaus H., Marks C., Wang Y., Anders N., Spiess A.C., Makowski L. (2016). Multi-scale processes of beech wood disintegration and pretreatment with 1-ethyl-3-methylimidazolium acetate/water mixtures. Biotechnol. Biofuels.

[82] Rinaldi R. (2011). Instantaneous dissolution of cellulose in organic electrolyte solutions. Chem. Commun..

[83] Xu A., Zhang Y., Zhao Y., Wang J. (2013). Cellulose dissolution at ambient temperature: Role of preferential solvation of cations of ionic liquids by a cosolvent. Carbohydr. Polym..

[84] Xu A., Guo X., Xu R. (2015). Understanding the dissolution of cellulose in 1-butyl-3-methylimidazolium acetate+ DMAc solvent. Int. J. Biol. Macromol..

[85] Xu A., Cao L., Wang B. (2015). Facile cellulose dissolution without heating in [C4mim][CH3COO]/DMF solvent. Carbohydr. Polym..

[86] Xu A., Chen L., Wang Y., Liu R., Niu W. (2019). Development of Diallylimidazolium Methoxyacetate/DMSO (DMF/DMA) Solvents for Improving Cellulose Dissolution and Fabricating Porous Material. Polymers.

[87] Yin W.P., Li X., Ren Y.L., Zhao S., Wang J.J. (2013). Selective hydrolysis of lignocelluloses from corn stalk in an ionic liquid. J. Appl. Polym. Sci..

[88] van Spronsen J., Cardoso M.A.T., Witkamp G.-J., de Jong W., Kroon M.C. (2011). Separation and recovery of the constituents from lignocellulosic biomass by using ionic liquids and acetic acid as co-solvents for mild hydrolysis. Chem. Eng. Process. Process Intensif..

[89] Xu A., Wang J., Wang H. (2010). Effects of anionic structure and lithium salts addition on the dissolution of cellulose in 1-butyl-3-methylimidazolium-based ionic liquid solvent systems. Green Chem..

[90] Pang Z., Dong C., Pan X. (2016). Enhanced deconstruction and dissolution of lignocellulosic biomass in ionic liquid at high water content by lithium chloride. Cellulose.

[91] Sun X., Sun X., Zhang F. (2016). Combined pretreatment of lignocellulosic biomass by solid base (calcined Na_2_SiO_3_) and ionic liquid for enhanced enzymatic saccharification. RSC Adv..

[92] Wei X., Wang Y., Li J., Wang F., Chang G., Fu T., Zhou W. (2018). Effects of temperature on cellulose hydrogen bonds during dissolution in ionic liquid. Carbohydr. Polym..

[93] Abushammala H. (2015). Novel Ionic Liquid-Mediated Technologies for the Extraction of Nanocellulose Directly from Wood. Ph.D. Thesis.

[94] Sun Y.-C., Xu J.-K., Xu F., Sun R.-C. (2013). Structural comparison and enhanced enzymatic hydrolysis of eucalyptus cellulose via pretreatment with different ionic liquids and catalysts. Process Biochem..

[95] Bahcegul E., Apaydin S., Haykir N.I., Tatli E., Bakir U. (2012). Different ionic liquids favor different lignocellulosic biomass particle sizes during pretreatment to function efficiently. Green Chem..

[96] Gschwend F.J., Chambon C.L., Biedka M., Brandt-Talbot A., Fennell P.S., Hallett J.P. (2019). Quantitative glucose release from softwood after pretreatment with low-cost ionic liquids. Green Chem..

[97] Sun Y.-C., Liu X.-N., Wang T.-T., Xue B.-L., Sun R.-C. (2019). Green Process for Extraction of Lignin by the Microwave-Assisted Ionic Liquid Approach: Toward Biomass Biorefinery and Lignin Characterization. ACS Sustain. Chem. Eng..

[98] Chen T., Li Y., Xu J., Hou Y. (2016). Dissolution of eucalyptus powder with alkaline ionic liquid [Mmim] DMP under microwave conditions. BioResources.

[99] Chen J., Jiang Q., Yang G., Wang Q., Fatehi P. (2017). Ultrasonic-assisted ionic liquid treatment of chemithermomechanical pulp fibers. Cellulose.

[100] Nakamura A., Miyafuji H., Saka S. (2010). Influence of reaction atmosphere on the liquefaction and depolymerization of wood in an ionic liquid, 1-ethyl-3-methylimidazolium chloride. J. Wood Sci..

[101] Abushammala H., Hashaikeh R. (2011). Enzymatic hydrolysis of cellulose and the use of TiO_2_ nanoparticles to open up the cellulose structure. Biomass Bioenergy.

[102] Casas A., Oliet M., Alonso M.V., Santos T.M., Rodriguez F. (2013). Dissolution of Pinus radiata and Eucalyptus globulus woods in 1-allyl-3-methylimidazolium chloride for cellulose or lignin regeneration. Ind. Eng. Chem. Res..

[103] Casas A., Alonso M., Oliet M., Santos T., Rodriguez F. (2013). Characterization of cellulose regenerated from solutions of pine and eucalyptus woods in 1-allyl-3-methilimidazolium chloride. Carbohydr. Polym..

[104] Crosthwaite J.M., Aki S.N., Maginn E.J., Brennecke J.F. (2005). Liquid phase behavior of imidazolium-based ionic liquids with alcohols: Effect of hydrogen bonding and non-polar interactions. Fluid Phase Equilibria.

[105] Liu H., Sale K.L., Simmons B.A., Singh S. (2011). Molecular dynamics study of polysaccharides in binary solvent mixtures of an ionic liquid and water. J. Phys. Chem. B.

[106] Li B., Asikkala J., Filpponen I., Argyropoulos D.S. (2010). Factors Affecting Wood Dissolution and Regeneration of Ionic Liquids. Ind. Eng. Chem. Res..

[107] Hashaikeh R., Abushammala H. (2011). Acid mediated networked cellulose: Preparation and characterization. Carbohydr. Polym..

[108] Halder P., Kundu S., Patel S., Parthasarathy R., Pramanik B., Paz-Ferreiro J., Shah K. (2019). TGA-FTIR study on the slow pyrolysis of lignin and cellulose-rich fractions derived from imidazolium-based ionic liquid pre-treatment of sugarcane straw. Energy Convers. Manag..

[109] da Costa Lopes A.M., João K.G., Rubik D.F., Bogel-Łukasik E., Duarte L.C., Andreaus J., Bogel-Łukasik R. (2013). Pre-treatment of lignocellulosic biomass using ionic liquids: Wheat straw fractionation. Bioresour. Technol..

[110] Hamidah U., Arakawa T., H’ng Y.Y., Nakagawa-izumi A., Kishino M. (2018). Recycled ionic liquid 1-ethyl-3-methylimidazolium acetate pretreatment for enhancing enzymatic saccharification of softwood without cellulose regeneration. J. Wood Sci..

[111] Viell J., Wulfhorst H., Schmidt T., Commandeur U., Fischer R., Spiess A., Marquardt W. (2013). An efficient process for the saccharification of wood chips by combined ionic liquid pretreatment and enzymatic hydrolysis. Bioresour. Technol..

[112] Fu D., Mazza G., Tamaki Y. (2010). Lignin extraction from straw by ionic liquids and enzymatic hydrolysis of the cellulosic residues. J. Agric. Food Chem..

[113] Yang H., Shi Z., Xiong L., Wang K., Sun R.-C. (2016). Ionic Liquids Assisted Alkaline Fractionation Enhanced Triploid Poplar Bioconversion for Bioethanol Production. Wood Res..

[114] Liu C.-Z., Wang F., Stiles A.R., Guo C. (2012). Ionic liquids for biofuel production: Opportunities and challenges. Appl. Energy.

[115] Kringstad K.P., Lindström K. (1984). Spent liquors from pulp bleaching. Environ. Sci. Technol..

[116] Thompson G., Swain J., Kay M., Forster C. (2001). The treatment of pulp and paper mill effluent: A review. Bioresour. Technol..

[117] Hu X., Cheng L., Gu Z., Hong Y., Li Z., Li C. (2018). Effects of ionic liquid/water mixture pretreatment on the composition, the structure and the enzymatic hydrolysis of corn stalk. Ind. Crop. Prod..

[118] Odorico F.H., de Araújo Morandim-Giannetti A., Lucarini A.C., Torres R.B. (2018). Pretreatment of Guinea grass (Panicum maximum) with the ionic liquid 1-ethyl-3-methyl imidazolium acetate for efficient hydrolysis and bioethanol production. Cellulose.

[119] Trinh L.T.P., Lee Y.-J., Lee J.-W., Lee W.-H. (2018). Optimization of ionic liquid pretreatment of mixed softwood by response surface methodology and reutilization of ionic liquid from hydrolysate. Biotechnol. Bioprocess Eng..

[120] Bose S., Barnes C.A., Petrich J.W. (2012). Enhanced stability and activity of cellulase in an ionic liquid and the effect of pretreatment on cellulose hydrolysis. Biotechnol. Bioeng..

[121] Wahlström R., Rovio S., Suurnäkki A. (2012). Partial enzymatic hydrolysis of microcrystalline cellulose in ionic liquids by Trichoderma reesei endoglucanases. RSC Adv..

[122] Xu J., Xiong P., He B. (2016). Advances in improving the performance of cellulase in ionic liquids for lignocellulose biorefinery. Bioresour. Technol..

[123] Financie R., Moniruzzaman M., Uemura Y. (2016). Enhanced enzymatic delignification of oil palm biomass with ionic liquid pretreatment. Biochem. Eng. J..

[124] Xie W.-H., Hu B.-B., Zhang X.-B., Yi X.-M., Tan W.-X., Zhu M.-J. (2015). Enhanced Enzymatic Digestibility of Sugarcane Bagasse Pretreated by Ionic Liquids. J. Biobased Mater. Bioenergy.

[125] Tian X., Rehmann L., Xu C.C., Fang Z. (2016). Pretreatment of eastern white pine (Pinus strobes L.) for enzymatic hydrolysis and ethanol production by organic electrolyte solutions. ACS Sustain. Chem. Eng..

[126] Zhang X., Zhao W., Li Y., Li C., Yuan Q., Cheng G. (2016). Synergistic effect of pretreatment with dimethyl sulfoxide and an ionic liquid on enzymatic digestibility of white poplar and pine. RSC Adv..

[127] Wu L., Lee S.-H., Endo T. (2013). Effect of dimethyl sulfoxide on ionic liquid 1-ethyl-3-methylimidazolium acetate pretreatment of eucalyptus wood for enzymatic hydrolysis. Bioresour. Technol..

[128] Fu D., Mazza G. (2011). Optimization of processing conditions for the pretreatment of wheat straw using aqueous ionic liquid. Bioresour. Technol..

[129] Fu D., Mazza G. (2011). Aqueous ionic liquid pretreatment of straw. Bioresour. Technol..

[130] Brandt A., Ray M.J., To T.Q., Leak D.J., Murphy R.J., Welton T. (2011). Ionic liquid pretreatment of lignocellulosic biomass with ionic liquid–water mixtures. Green Chem..

[131] Funazukuri T., Ozawa S. (2019). Effects of Pretreatment with Ionic Liquids on Cellulose Hydrolysis under Hydrothermal Conditions. Molecules.

[132] Rigual V., Domínguez J.C., Santos T.M., Rivas S., Alonso M.V., Oliet M., Rodriguez F. (2019). Autohydrolysis and microwave ionic liquid pretreatment of Pinus radiata: Imaging visualization and analysis to understand enzymatic digestibility. Ind. Crop. Prod..

[133] Rigual V., Santos T.M., Domínguez J.C., Alonso M.V., Oliet M., Rodriguez F. (2018). Combining autohydrolysis and ionic liquid microwave treatment to enhance enzymatic hydrolysis of Eucalyptus globulus wood. Bioresour. Technol..

[134] Elmacı S.B., Özçelik F. (2018). Ionic liquid pretreatment of yellow pine followed by enzymatic hydrolysis and fermentation. Biotechnol. Prog..

[135] Dotsenko A.S., Dotsenko G.S., Senko O.V., Stepanov N.A., Lyagin I.V., Efremenko E.N., Gusakov A.V., Zorov I.N., Rubtsova E.A. (2018). Complex effect of lignocellulosic biomass pretreatment with 1-butyl-3-methylimidazolium chloride ionic liquid on various aspects of ethanol and fumaric acid production by immobilized cells within SSF. Bioresour. Technol..

[136] Ninomiya K., Ochiai K., Eguchi M., Kuroda K., Tsuge Y., Ogino C., Taima T., Takahashi K. (2018). Oxidative depolymerization potential of biorefinery lignin obtained by ionic liquid pretreatment and subsequent enzymatic saccharification of eucalyptus. Ind. Crop. Prod..

[137] Goshadrou A., Lefsrud M. (2017). Synergistic surfactant-assisted [EMIM] OAc pretreatment of lignocellulosic waste for enhanced cellulose accessibility to cellulase. Carbohydr. Polym..

[138] Hashmi M., Sun Q., Tao J., Wells Jr T., Shah A.A., Labbe N., Ragauskas A.J. (2017). Comparison of autohydrolysis and ionic liquid 1-butyl-3-methylimidazolium acetate pretreatment to enhance enzymatic hydrolysis of sugarcane bagasse. Bioresour. Technol..

[139] Chen S., Zhang X., Ling Z., Xu F. (2017). Characterization of the micromorphology and topochemistry of poplar wood during mild ionic liquid pretreatment for improving enzymatic saccharification. Molecules.

[140] Aid T., Hyvärinen S., Vaher M., Koel M., Mikkola J.-P. (2016). Saccharification of lignocellulosic biomasses via ionic liquid pretreatment. Ind. Crop. Prod..

[141] Vo H.T., Kim C.S., Lee S.D., Lee H. (2016). Ionic Liquid-assisted Separation of Carbohydrates from Lignocellulosic Biomass. Bull. Korean Chem. Soc..

[142] Normark M., Pommer L., Gräsvik J., Hedenström M., Gorzsás A., Winestrand S., Jönsson L.J. (2016). Biochemical conversion of torrefied Norway spruce after pretreatment with acid or ionic liquid. Bioenergy Res..

[143] Torr K.M., Love K.T., Simmons B.A., Hill S.J. (2016). Structural features affecting the enzymatic digestibility of pine wood pretreated with ionic liquids. Biotechnol. Bioeng..

[144] Wang S., You T., Xu F., Chen J., Yang G. (2015). Optimization of [Amim] Cl pretreatment conditions for maximum glucose recovery from hybrid Pennisetum by response surface methodology. BioResources.

[145] Trinh L.T.P., Lee Y.J., Lee J.-W., Lee H.-J. (2015). Characterization of ionic liquid pretreatment and the bioconversion of pretreated mixed softwood biomass. Biomass Bioenergy.

[146] Auxenfans T., Buchoux S., Larcher D., Husson G., Husson E., Sarazin C. (2014). Enzymatic saccharification and structural properties of industrial wood sawdust: Recycled ionic liquids pretreatments. Energy Convers. Manag..

[147] Yáñez R., Gómez B., Martínez M., Gullón B., Alonso J.L. (2014). Valorization of an invasive woody species, Acacia dealbata, by means of Ionic liquid pretreatment and enzymatic hydrolysis. J. Chem. Technol. Biotechnol..

[148] Bian J., Peng F., Peng X.-P., Xiao X., Peng P., Xu F., Sun R.-C. (2014). Effect of [Emim] Ac pretreatment on the structure and enzymatic hydrolysis of sugarcane bagasse cellulose. Carbohydr. Polym..

[149] Cui L.H., Wang M., Li J.H., Wang Q.H. (2014). Effect of Ionic Liquid Pretreatment on the Structure and Enzymatic Saccharification of Cassava Stillage Residues. Adv. Mater. Res..

[150] Soudham V.P., Gräsvik J., Alriksson B., Mikkola J.P., Jönsson L.J. (2013). Enzymatic hydrolysis of Norway spruce and sugarcane bagasse after treatment with 1-allyl-3-methylimidazolium formate. J. Chem. Technol. Biotechnol..

[151] Wang J., Wang H., Mou H. (2013). 1-allyl-3-methylimidazolium chloride pretreatment of seaweed industrial waste for bioethanol conversion. J. Renew. Sustain. Energy.

[152] Haykir N.I., Bakir U. (2013). Ionic liquid pretreatment allows utilization of high substrate loadings in enzymatic hydrolysis of biomass to produce ethanol from cotton stalks. Ind. Crop. Prod..

[153] Yuan T.-Q., Wang W., Xu F., Sun R.-C. (2013). Synergistic benefits of ionic liquid and alkaline pretreatments of poplar wood. Part 1: Effect of integrated pretreatment on enzymatic hydrolysis. Bioresour. Technol..

[154] Mou H.-Y., Orblin E., Kruus K., Fardim P. (2013). Topochemical pretreatment of wood biomass to enhance enzymatic hydrolysis of polysaccharides to sugars. Bioresour. Technol..

[155] Goshadrou A., Karimi K., Lefsrud M. (2013). Characterization of ionic liquid pretreated aspen wood using semi-quantitative methods for ethanol production. Carbohydr. Polym..

[156] Socha A.M., Plummer S.P., Stavila V., Simmons B.A., Singh S. (2013). Comparison of sugar content for ionic liquid pretreated Douglas-fir woodchips and forestry residues. Biotechnol. Biofuels.

[157] Li C., Sun L., Simmons B.A., Singh S. (2013). Comparing the recalcitrance of eucalyptus, pine, and switchgrass using ionic liquid and dilute acid pretreatments. Bioenergy Res..

[158] Shafiei M., Zilouei H., Zamani A., Taherzadeh M.J., Karimi K. (2013). Enhancement of ethanol production from spruce wood chips by ionic liquid pretreatment. Appl. Energy.

[159] Lee K.M., Ngoh G.C., Chua A.S.M. (2013). Process optimization and performance evaluation on sequential ionic liquid dissolution–solid acid saccharification of sago waste. Bioresour. Technol..

[160] Tan H.T., Lee K.T. (2012). Understanding the impact of ionic liquid pretreatment on biomass and enzymatic hydrolysis. Chem. Eng. J..

[161] Ninomiya K., Kamide K., Takahashi K., Shimizu N. (2012). Enhanced enzymatic saccharification of kenaf powder after ultrasonic pretreatment in ionic liquids at room temperature. Bioresour. Technol..

[162] da Silva A.S.A., Lee S.-H., Endo T., Bon E.P. (2011). Major improvement in the rate and yield of enzymatic saccharification of sugarcane bagasse via pretreatment with the ionic liquid 1-ethyl-3-methylimidazolium acetate ([Emim][Ac]). Bioresour. Technol..

[163] Kimon K.S., Alan E.L., Sinclair D.W.O. (2011). Enhanced saccharification kinetics of sugarcane bagasse pretreated in 1-butyl-3-methylimidazolium chloride at high temperature and without complete dissolution. Bioresour. Technol..

[164] Holmbom B. (1980). A procedure for analysis of toxic compounds in pulp and paper mill waste waters. Pap. Puu (Pap. Pulp).

[165] Servos M.R. (1996). Environmental Fate and Effects of Pulp and Paper: Mill Effluents.

[166] Zhu J., Chai X.-S., Pan X., Luo Q., Li J. (2002). Quantification and reduction of organic sulfur compound formation in a commercial wood pulping process. Environ. Sci. Technol..

[167] Kim J.-Y., Shin E.-J., Eom I.-Y., Won K., Kim Y.H., Choi D., Choi I.-G., Choi J.W. (2011). Structural features of lignin macromolecules extracted with ionic liquid from poplar wood. Bioresour. Technol..

[168] da Silva S.P.M., da Costa Lopes A.M., Roseiro L.B., Bogel-Łukasik R. (2013). Novel pre-treatment and fractionation method for lignocellulosic biomass using ionic liquids. RSC Adv..

[169] Lan W., Liu C.-F., Sun R.-C. (2011). Fractionation of Bagasse into Cellulose, Hemicelluloses, and Lignin with Ionic Liquid Treatment Followed by Alkaline Extraction. J. Agric. Food Chem..

[170] Penín L., Peleteiro S., Rivas S., Santos V., Parajo J.C. (2019). Production of 5-Hydroxymethylfurfural from Pine Wood via Biorefinery Technologies Based on Fractionation and Reaction in Ionic Liquids. BioResources.

[171] Simmons T.J., Lee S.H., Miao J., Miyauchi M., Park T.-J., Bale S.S., Pangule R., Bult J., Martin J.G., Dordick J.S. (2011). Preparation of synthetic wood composites using ionic liquids. Wood Sci. Technol..

[172] Barta K., Warner G.R., Beach E.S., Anastas P.T. (2014). Depolymerization of organosolv lignin to aromatic compounds over Cu-doped porous metal oxides. Green Chem..

[173] Younesi-Kordkheili H., Pizzi A. (2018). Properties of plywood panels bonded with ionic liquid-modified lignin–phenol–formaldehyde resin. J. Adhes..

[174] Prado R., Erdocia X., Labidi J. (2013). Lignin extraction and purification with ionic liquids. J. Chem. Technol. Biotechnol..

[175] Xu J.-K., Sun Y.-C., Xu F., Sun R.-C. (2013). Characterization of hemicelluloses obtained from partially delignified Eucalyptus using ionic liquid pretreatment. BioResources.

[176] Sixta H., Iakovlev M., Testova L., Roselli A., Hummel M., Borrega M., van Heiningen A., Froschauer C., Schottenberger H. (2013). Novel concepts of dissolving pulp production. Cellulose.

[177] Roselli A., Hummel M., Monshizadeh A., Maloney T., Sixta H. (2014). Ionic liquid extraction method for upgrading eucalyptus kraft pulp to high purity dissolving pulp. Cellulose.

[178] Froschauer C., Hummel M., Iakovlev M., Roselli A., Schottenberger H., Sixta H. (2013). Separation of hemicellulose and cellulose from wood pulp by means of ionic liquid/cosolvent systems. Biomacromolecules.

[179] Abushammala H., Goldsztayn R., Leao A., Laborie M.-P. (2016). Combining steam explosion with 1-ethyl-3-methylimidazlium acetate treatment of wood yields lignin-coated cellulose nanocrystals of high aspect ratio. Cellulose.

[180] Abushammala H., Krossing I., Laborie M.-P. (2015). Ionic liquid-mediated technology to produce cellulose nanocrystals directly from wood. Carbohydr. Polym..

[181] Janesko B.G. (2011). Modeling interactions between lignocellulose and ionic liquids using DFT-D. Phys. Chem. Chem. Phys..

[182] Payal R.S., Bejagam K.K., Mondal A., Balasubramanian S. (2015). Dissolution of Cellulose in Room Temperature Ionic Liquids: Anion Dependence. J. Phys. Chem. B.

[183] Guo J., Zhang D., Duan C., Liu C. (2010). Probing anion–cellulose interactions in imidazolium-based room temperature ionic liquids: A density functional study. Carbohydr. Res..

[184] George A., Tran K., Morgan T.J., Benke P.I., Berrueco C., Lorente E., Wu B.C., Keasling J.D., Simmons B.A., Holmes B.M. (2011). The effect of ionic liquid cation and anion combinations on the macromolecular structure of lignins. Green Chem..

[185] Nakamura A., Miyafuji H., Saka S. (2010). Liquefaction behavior of Western red cedar and Japanese beech in the ionic liquid 1-ethyl-3-methylimidazolium chloride. Holzforschung.

[186] Moniruzzaman M., Ono T. (2013). Separation and characterization of cellulose fibers from cypress wood treated with ionic liquid prior to laccase treatment. Bioresour. Technol..

[187] Shamsuri A.A., Abdullah D.K. (2010). Isolation and characterization of lignin from rubber wood in ionic liquid medium. Mod. Appl. Sci..

[188] Ladesov A., Belesov A., Kuznetsova M., Pochtovalova A., Malkov A., Shestakov S., Kosyakov D. (2018). Fractionation of Wood with Binary Solvent 1-Butyl-3-methylimidazolium Acetate+ Dimethyl Sulfoxide. Russ. J. Appl. Chem..

[189] Wen J.-L., Sun S.-L., Xue B.-L., Sun R.-C. (2013). Quantitative structures and thermal properties of birch lignins after ionic liquid pretreatment. J. Agric. Food Chem..

[190] Tan S.S.Y., MacFarlane D.R., Upfal J., Edye L.A., Doherty W.O.S., Patti A.F., Pringle J.M., Scott J.L. (2009). Extraction of lignin from lignocellulose at atmospheric pressure using alkylbenzenesulfonate ionic liquid. Green Chem..

[191] El-barbary M.H., Shukry N. (2008). Polyhydric alcohol liquefaction of some lignocellulosic agricultural residues. Ind. Crop. Prod..

[192] Alma M., Yoshioka M., Yao Y., Shiraishi N. (1995). Preparation and characterization of the phenolated wood using hydrochloric acid (HCl) as a catalyst. Wood Sci. Technol..

[193] Xie H., King A., Kilpelainen I., Granstrom M., Argyropoulos D.S. (2007). Thorough chemical modification of wood-based lignocellulosic materials in ionic liquids. Biomacromolecules.

[194] Ou R., Xie Y., Wang Q., Sui S., Wolcott M.P. (2014). Thermoplastic deformation of poplar wood plasticized by ionic liquids measured by a nonisothermal compression technique. Holzforschung.

[195] Brandt A., Erickson J.K., Hallett J.P., Murphy R.J., Potthast A., Ray M.J., Rosenau T., Schrems M., Welton T. (2012). Soaking of pine wood chips with ionic liquids for reduced energy input during grinding. Green Chem..

[196] Kanbayashi T., Miyafuji H. (2016). Influence of ionic liquid treatment on wood cell walls: Anatomical changes in opposite wood. Eur. J. Wood Wood Prod..

[197] Lucas M., Wagner G.L., Nishiyama Y., Hanson L., Samayam I.P., Schall C.A., Langan P., Rector K.D. (2011). Reversible swelling of the cell wall of poplar biomass by ionic liquid at room temperature. Bioresour. Technol..

[198] Ou R., Xie Y., Wang Q., Sui S., Wolcott M.P. (2015). Material pocket dynamic mechanical analysis: A novel tool to study thermal transition in wood fibers plasticized by an ionic liquid (IL). Holzforschung.

[199] Salmén L. (1984). Viscoelastic properties ofin situ lignin under water-saturated conditions. J. Mater. Sci..

[200] Goring D.A. (1963). Thermal softening of lignin, hemicelluolose and cellulose. Pulp Pap.

[201] Blechschmidt J., Engert P., Stephan M. (1986). The glass transition of wood from the viewpoint of mechanical pulping. Wood Sci. Technol..

[202] Eriksson I., Haglind I., Lidbrandt O., Sahnén L. (1991). Fiber swelling favoured by lignin softening. Wood Sci. Technol..

[203] Zhang K., Xiao H., Su Y., Wu Y., Cui Y., Li M. (2019). Mechanical and Physical Properties of Regenerated Biomass Composite Films from Lignocellulosic Materials in Ionic Liquid. BioResources.

[204] Adak B., Mukhopadhyay S. (2016). Effect of the dissolution time on the structure and properties of lyocell-fabric-based all-cellulose composite laminates. J. Appl. Polym. Sci..

[205] Shu Z., Song J., Yuan Y., Chen J., Zhang S., Huang L., Liu S. (2018). Preparation and Mechanical Properties of Lignocellulosic Composite Films Based on Poplar Wood Flour and Waste Filter Paper. BioResources.

[206] Shibata M., Teramoto N., Nakamura T., Saitoh Y. (2013). All-cellulose and all-wood composites by partial dissolution of cotton fabric and wood in ionic liquid. Carbohydr. Polym..

[207] Mahmood H., Moniruzzaman M., Yusup S., Akil H.M. (2018). Ionic liquid pretreatment at high solids loading: A clean approach for fabrication of renewable resource based particulate composites. Polym. Compos..

[208] Li J., Zhang X., Zhang J., Mi Q., Jia F., Wu J., Yu J., Zhang J. (2019). Direct and complete utilization of agricultural straw to fabricate all-biomass films with high-strength, high-haze and UV-shielding properties. Carbohydr. Polym..

[209] Khakalo A., Tanaka A., Korpela A., Hauru L.K., Orelma H. (2019). All-Wood Composite Material by Partial Fiber Surface Dissolution with an Ionic Liquid. ACS Sustain. Chem. Eng..

[210] Tanaka A., Khakalo A., Hauru L., Korpela A., Orelma H. (2018). Conversion of paper to film by ionic liquids: Manufacturing process and properties. Cellulose.

[211] Khosravani A., Pourjafar M., Behrooz R. The effect of lignin on processing and the properties of lignocellulose material recovered by ionic liquid. Proceedings of the IOP Conference Series: Materials Science and Engineering.

[212] Tisserat B., Larson E., Gray D., Dexter N., Meunier C., Moore L., Haverhals L. (2015). Ionic liquid-facilitated preparation of lignocellulosic composites. Int. J. Polym. Sci..

[213] Shibata M., Yamazoe K., Kuribayashi M., Okuyama Y. (2013). All-wood biocomposites by partial dissolution of wood flour in 1-butyl-3-methylimidazolium chloride. J. Appl. Polym. Sci..

[214] Abushammala H., Mao J. (2019). A Review of the Surface Modification of Cellulose and Nanocellulose Using Aliphatic and Aromatic Mono-and Di-Isocyanates. Molecules.

[215] Dufresne A. (2013). Nanocellulose: A new ageless bionanomaterial. Mater. Today.

[216] Klemm D., Cranston E.D., Fischer D., Gama M., Kedzior S.A., Kralisch D., Kramer F., Kondo T., Lindström T., Nietzsche S. (2018). Nanocellulose as a natural source for groundbreaking applications in materials science: Today’s state. Mater. Today.

[217] Eyley S., Thielemans W. (2014). Surface modification of cellulose nanocrystals. Nanoscale.

[218] Kiziltas A., Kiziltas E.E., Boran S., Gardner D.J. Micro-and nanocellulose composites for automotive applications. Proceedings of the SPE Automotive Composites Conference and Exhibition (ACCE).

[219] Voisin H., Bergström L., Liu P., Mathew A.P. (2017). Nanocellulose-based materials for water purification. Nanomaterials.

[220] Sharma P.R., Chattopadhyay A., Sharma S.K., Geng L., Amiralian N., Martin D., Hsiao B.S. (2018). Nanocellulose from Spinifex as an Effective Adsorbent to Remove Cadmium(II) from Water. ACS Sustain. Chem. Eng..

[221] Pirani S., Abushammala H.M., Hashaikeh R. (2013). Preparation and characterization of electrospun PLA/nanocrystalline cellulose-based composites. J. Appl. Polym. Sci..

[222] Abushammala H., Hashaikeh R., Cooney C. (2012). Microcrystalline cellulose powder tableting via networked cellulose-based gel material. Powder Technol..

[223] Shi Z., Phillips G.O., Yang G. (2013). Nanocellulose electroconductive composites. Nanoscale.

[224] (2017). Standard Terms and Their Definition for Cellulose Nanomaterial.

[225] Li C., Zhao Z.K. (2007). Efficient acid-catalyzed hydrolysis of cellulose in ionic liquid. Adv. Synth. Catal..

[226] Bondeson D., Mathew A., Oksman K. (2006). Optimization of the isolation of nanocrystals from microcrystalline cellulose by acid hydrolysis. Cellulose.

[227] Fan J.-S., Li Y.-H. (2012). Maximizing the yield of nanocrystalline cellulose from cotton pulp fiber. Carbohydr. Polym..

[228] Reyes G., Aguayo M.G., Fernández Pérez A., Pääkkönen T., Gacitúa W., Rojas O.J. (2019). Dissolution and Hydrolysis of Bleached Kraft Pulp Using Ionic Liquids. Polymers.

[229] Man Z., Muhammad N., Sarwono A., Bustam M.A., Kumar M.V., Rafiq S. (2011). Preparation of cellulose nanocrystals using an ionic liquid. J. Polym. Environ..

[230] Mao J., Osorio-Madrazo A., Laborie M.-P. (2013). Preparation of cellulose I nanowhiskers with a mildly acidic aqueous ionic liquid: Reaction efficiency and whiskers attributes. Cellulose.

[231] Mao J., Abushammala H., Pereira L.B., Laborie M.-P. (2016). Swelling and hydrolysis kinetics of Kraft pulp fibers in aqueous 1-butyl-3-methylimidazolium hydrogen sulfate solutions. Carbohydr. Polym..

[232] Grząbka-Zasadzińska A., Skrzypczak A., Borysiak S. (2019). The influence of the cation type of ionic liquid on the production of nanocrystalline cellulose and mechanical properties of chitosan-based biocomposites. Cellulose.

[233] Han J., Zhou C., French A.D., Han G., Wu Q. (2013). Characterization of cellulose II nanoparticles regenerated from 1-butyl-3-methylimidazolium chloride. Carbohydr. Polym..

[234] Mao J., Heck B., Abushammala H., Reiter G., Laborie M.-P. (2019). A structural fibrillation parameter from small angle X-ray scattering to quantify pulp refining. Cellulose.

[235] Turbak A.F., Snyder F.W., Sandberg K.R. (1983). Microfibrillated cellulose, a new cellulose product: Properties, uses, and commercial potential. J. Appl. Polym. Sci. Appl. Polym. Symp..

[236] Nair S.S., Zhu J., Deng Y., Ragauskas A.J. (2014). Characterization of cellulose nanofibrillation by micro grinding. J. Nanoparticle Res..

[237] Sharma P.R., Joshi R., Sharma S.K., Hsiao B.S. (2017). A Simple Approach to Prepare Carboxycellulose Nanofibers from Untreated Biomass. Biomacromolecules.

[238] Viswanathan G., Murugesan S., Pushparaj V., Nalamasu O., Ajayan P.M., Linhardt R.J. (2006). Preparation of biopolymer fibers by electrospinning from room temperature ionic liquids. Biomacromolecules.

[239] Ahn Y., Hu D.-H., Hong J.H., Lee S.H., Kim H.J., Kim H. (2012). Effect of co-solvent on the spinnability and properties of electrospun cellulose nanofiber. Carbohydr. Polym..

[240] Mao J., Abushammala H., Hettegger H., Rosenau T., Laborie M.-P. (2017). Imidazole, a New Tunable Reagent for Producing Nanocellulose, Part I: Xylan-Coated CNCs and CNFs. Polymers.

[241] Laborie M.P., Abushammala H. (2018). Ionic-Liquid Mediated Production of Cellulose Nanocrystals Directly from Wood, Grass or Bioresidues. U.S. Patent.

[242] Rajinipriya M., Nagalakshmaiah M., Robert M., Elkoun S. (2018). Importance of Agricultural and Industrial Waste in the Field of Nanocellulose and Recent Industrial Developments of Wood Based Nanocellulose: A Review. ACS Sustain. Chem. Eng..

[243] Liu C., Li Y., Hou Y. (2018). Preparation and structural characterization of lignin micro/nano-particles with ionic liquid treatment by self-assembly. Express Polym. Lett..

[244] Karatzos S.K., Edye L.A., Wellard R.M. (2012). The undesirable acetylation of cellulose by the acetate ion of 1-ethyl-3-methylimidazolium acetate. Cellulose.

[245] Çetinkol Ö.P., Dibble D.C., Cheng G., Kent M.S., Knierim B., Auer M., Wemmer D.E., Pelton J.G., Melnichenko Y.B., Ralph J. (2010). Understanding the impact of ionic liquid pretreatment on eucalyptus. Biofuels.

[246] Zweckmair T., Hettegger H., Abushammala H., Bacher M., Potthast A., Laborie M.-P., Rosenau T. (2015). On the mechanism of the unwanted acetylation of polysaccharides by 1, 3-dialkylimidazolium acetate ionic liquids: Part 1—Analysis, acetylating agent, influence of water, and mechanistic considerations. Cellulose.

[247] Köhler S., Liebert T., Schöbitz M., Schaller J., Meister F., Günther W., Heinze T. (2007). Interactions of Ionic Liquids with Polysaccharides 1. Unexpected Acetylation of Cellulose with 1-Ethyl-3-methylimidazolium Acetate. Macromol. Rapid Commun..

[248] Abushammala H., Hettegger H., Bacher M., Korntner P., Potthast A., Rosenau T., Laborie M.-P. (2017). On the mechanism of the unwanted acetylation of polysaccharides by 1, 3-dialkylimidazolium acetate ionic liquids: Part 2—The impact of lignin on the kinetics of cellulose acetylation. Cellulose.

[249] Hauru L.K., Ma Y., Hummel M., Alekhina M., King A.W., Kilpeläinen I., Penttilä P.A., Serimaa R., Sixta H. (2013). Enhancement of ionic liquid-aided fractionation of birchwood. Part 1: Autohydrolysis pretreatment. RSC Adv..

[250] Ohno E., Miyafuji H. (2013). Reaction behavior of cellulose in an ionic liquid, 1-ethyl-3-methylimidazolium chloride. J. Wood Sci..

[251] Leskinen T., King A.W., Argyropoulos D.S. (2014). Fractionation of lignocellulosic materials with ionic liquids. Production of Biofuels and Chemicals with Ionic Liquids.

[252] King A.W., Xie H., Fiskari J., Kilpelaeinen I. (2014). Chapter 5: Reduction of Biomass Recalcitrance via Ionic Liquid Pretreatments. Materials for Biofuels.

[253] Gazit O.M., Katz A. (2012). Dialkylimidazolium ionic liquids hydrolyze cellulose under mild conditions. ChemSusChem.

[254] Ebner G., Schiehser S., Potthast A., Rosenau T. (2008). Side reaction of cellulose with common 1-alkyl-3-methylimidazolium-based ionic liquids. Tetrahedron Lett..

[255] Handy S.T., Okello M. (2005). The 2-position of imidazolium ionic liquids: Substitution and exchange. J. Org. Chem..

[256] Clough M.T., Geyer K., Hunt P.A., Son S., Vagt U., Welton T. (2015). Ionic liquids: Not always innocent solvents for cellulose. Green Chem..

[257] Du H., Qian X. (2011). The effects of acetate anion on cellulose dissolution and reaction in imidazolium ionic liquids. Carbohydr. Res..

[258] Cremer T., Kolbeck C., Lovelock K.R., Paape N., Wölfel R., Schulz P.S., Wasserscheid P., Weber H., Thar J., Kirchner B. (2010). Towards a Molecular Understanding of Cation–Anion Interactions—Probing the Electronic Structure of Imidazolium Ionic Liquids by NMR Spectroscopy, X-ray Photoelectron Spectroscopy and Theoretical Calculations. Chem. A Eur. J..

[259] Rodríguez H., Gurau G., Holbrey J.D., Rogers R.D. (2011). Reaction of elemental chalcogens with imidazolium acetates to yield imidazole-2-chalcogenones: Direct evidence for ionic liquids as proto-carbenes. Chem. Commun..

[260] Abushammala H., Winter H., Krossing I., Laborie M.-P. (2014). On the prevalence of side reactions during ionosolv pulping of Norway spruce with 1-butyl-3-methylimidazolium acesulfamate. Cellulose.

[261] Peleteiro S., Santos V., Garrote G., Parajó J.C. (2016). Furfural production from Eucalyptus wood using an acidic ionic liquid. Carbohydr. Polym..

[262] Ito R., Miyafuji H., Miyazaki Y., Kawai T. (2016). Production of 5-hydroxymethylfurfural from wood by ionic liquid treatment. J. Wood Sci..

[263] Li W., Sun N., Stoner B., Jiang X., Lu X., Rogers R.D. (2011). Rapid dissolution of lignocellulosic biomass in ionic liquids using temperatures above the glass transition of lignin. Green Chem..

[264] Rigual V., Santos T.M., Domínguez J.C., Alonso M.V., Oliet M., Rodriguez F. (2017). Recovery and Reuse of 1-Allyl-3-methylimidazolium Chloride in the Fractionation of Pinus radiata Wood. ACS Sustain. Chem. Eng..

[265] Ohno E., Miyafuji H. (2014). Decomposition of cellulose in an ionic liquid, 1-ethyl-3-methylimidazolium chloride. J. Wood Sci..

[266] Brennan T.C., Datta S., Blanch H.W., Simmons B.A., Holmes B.M. (2010). Recovery of sugars from ionic liquid biomass liquor by solvent extraction. Bioenergy Res..

[267] Lipscomb G., Varanasi S., Paripati P., Dadi A.P. (2012). Ionic Liquid Recovery and Purification in Biomass Treatment Processes. W.O. Patent.

[268] Abu-Eishah S.I. (2011). Ionic liquids recycling for reuse. Ion. Liq. Cl. Prop..

[269] Wohlfarth C. (2009). Viscosity of Pure Organic Liquids and Binary Liquid Mixtures.

[270] Sigma-Aldrich Website. www.sigmaaldrich.com.

[271] TCI Chemicals Website. www.tcichemicals.com.

[272] Alfa Aesar Website. www.alfa.com.

[273] Acros Organics Website. www.acros.com.

[274] Stepnowski P., Mrozik W., Nichthauser J. (2007). Adsorption of alkylimidazolium and alkylpyridinium ionic liquids onto natural soils. Environ. Sci. Technol..

[275] Pham T.P.T., Cho C.-W., Yun Y.-S. (2010). Environmental fate and toxicity of ionic liquids: A review. Water Res..

[276] Docherty K.M., Hebbeler S.Z., Kulpa C.F. (2006). An assessment of ionic liquid mutagenicity using the Ames Test. Green Chem..

[277] Hassoun E., Abraham M., Kini V., Al-Ghafri M., Abushaban A. (2002). Cytotoxicity of the ionic liquid, 1-N-butyl-3-methyl imidazolium chloride. Res. Commun. Pharmacol. Toxicol..

[278] Pretti C., Chiappe C., Pieraccini D., Gregori M., Abramo F., Monni G., Intorre L. (2006). Acute toxicity of ionic liquids to the zebrafish (Danio rerio). Green Chem..

[279] Wells A.S., Coombe V.T. (2006). On the freshwater ecotoxicity and biodegradation properties of some common ionic liquids. Org. Process Res. Dev..

[280] Bernot R.J., Brueseke M.A., Evans-White M.A., Lamberti G.A. (2005). Acute and chronic toxicity of imidazolium-based ionic liquids on Daphnia magna. Environ. Toxicol. Chem. Int. J..

[281] Docherty K.M., Kulpa C.F. (2005). Toxicity and antimicrobial activity of imidazolium and pyridinium ionic liquids. Green Chem..

[282] Pernak J., Sobaszkiewicz K., Mirska I. (2003). Anti-microbial activities of ionic liquids. Green Chem..

[283] Pernak J., Goc I., Mirska I. (2004). Anti-microbial activities of protic ionic liquids with lactate anion. Green Chem..

[284] Stolte S., Arning J., Bottin-Weber U., Matzke M., Stock F., Thiele K., Uerdingen M., Welz-Biermann U., Jastorff B., Ranke J. (2006). Anion effects on the cytotoxicity of ionic liquids. Green Chem..

[285] Latała A., Stepnowski P., Nędzi M., Mrozik W. (2005). Marine toxicity assessment of imidazolium ionic liquids: Acute effects on the Baltic algae Oocystis submarina and Cyclotella meneghiniana. Aquat. Toxicol..

[286] Righi S., Morfino A., Galletti P., Samorì C., Tugnoli A., Stramigioli C. (2011). Comparative cradle-to-gate life cycle assessments of cellulose dissolution with 1-butyl-3-methylimidazolium chloride and N-methyl-morpholine-N-oxide. Green Chem..

[287] Kahani S., Shafiei M., Abdolmaleki A., Karimi K. (2017). Enhancement of ethanol production by novel morpholinium ionic liquids. J. Clean. Prod..

[288] Ninomiya K., Abe M., Tsukegi T., Kuroda K., Omichi M., Takada K., Noguchi M., Tsuge Y., Ogino C., Taki K. (2017). Ionic liquid pretreatment of bagasse improves mechanical property of bagasse/polypropylene composites. Ind. Crop. Prod..

[289] Ninomiya K., Ohta A., Omote S., Ogino C., Takahashi K., Shimizu N. (2013). Combined use of completely bio-derived cholinium ionic liquids and ultrasound irradiation for the pretreatment of lignocellulosic material to enhance enzymatic saccharification. Chem. Eng. J..

[290] Hamada Y., Yoshida K., Asai R.-I., Hayase S., Nokami T., Izumi S., Itoh T. (2013). A possible means of realizing a sacrifice-free three component separation of lignocellulose from wood biomass using an amino acid ionic liquid. Green Chem..

[291] Muhammad N., Man Z., Mutalib M.A., Bustam M.A., Wilfred C.D., Khan A.S., Ullah Z., Gonfa G., Nasrullah A. (2015). Dissolution and separation of wood biopolymers using ionic liquids. Chembioeng Rev..

